# Nutraceutical potential of moroccan *Juglans regia*: phytochemical profiling and multi-modal evaluation of bioactivities

**DOI:** 10.3389/fchem.2025.1638205

**Published:** 2025-09-04

**Authors:** Aziz Drioiche, Omkulthom Al kamaly, Soukayna Baammi, Khalid Zibouh, Rachid Amaiach, Laila Bennani, El Ouali Lalami Abdelhakim, Ahde El Imache, Fadoua EL Makhoukhi, Sevser Sahpaz, Touriya Zair

**Affiliations:** 1 Higher Institute of Nursing and Health Techniques of Fez, Regional Health Directorate Fez-Meknes, El Ghassani Hospital, Fez, Morocco; 2 Research Team of Chemistry of Bioactive Molecules and the Environment, Laboratory of Innovative Materials and Biotechnology of Natural Resources, Faculty of Sciences, Moulay Ismaïl University, Meknes, Morocco; 3 Department of Pharmaceutical Sciences, College of Pharmacy, Princess Nourah bint Abdulrahman University, Riyadh, Saudi Arabia; 4 Bioinformatics Laboratory, College of Computing, Mohammed VI Polytechnic University, Ben Guerir, Morocco; 5 Laboratory of Innovative Technologies, Process Engineering Department, Higher School of Technology Fez, USMBA, Fez, Morocco; 6 Laboratory of Spectroscopy, Molecular Modelling, Materials, Nanomaterial, Water and Environment, CERNE2D, Faculty of Science, Mohammed V University in Rabat, Rabat, Morocco; 7 Univ. Lille, University of Liège, University of Picardie Jules Verne, JUNIA, UMRT 1158 BioEcoAgro, Specialized Metabolites of Plant Origin, Lille, France

**Keywords:** *Juglans regia* L., pedunculagin, hydrojuglone glucoside, DPPH, total antioxidant capacity, FRAP, anticoagulant activity, antimicrobial activity

## Abstract

This study explores the therapeutic potential of *J. regia* from Morocco through an in-depth phytochemical analysis and biological evaluations using *in vitro*, *in vivo*, and *in silico* approaches. The extracts of *J. regia* were obtained through decoction and Soxhlet extraction, utilizing water and a water-ethanol mixture as extraction solvents. Phenolic compounds, flavonoids, condensed tannins, and hydrolyzable tannins were quantified using spectrophotometric methods. Chromatographic analysis by HPLC/MS enabled the identification and characterization of the main bioactive compounds. Biological activities were assessed through antioxidant (DPPH, FRAP, TAC), antimicrobial (MIC, MBC), anticoagulant (prothrombin time and activated partial thromboplastin time), and antidiabetic (inhibition of α-amylase and α-glucosidase enzymes, *in vivo* tests) assays. *In silico* simulations were conducted to study the molecular interactions between the significant compounds and their biological targets. The extracts, obtained by decoction and Soxhlet extraction, revealed a richness in bioactive compounds, notably pedunculagin (45.12%), hydrojuglone glucoside (14.51%), and gallic acid (5.18%), with high concentrations of polyphenols (42.105 mg GAE/g) and flavonoids (14.888 mg QE/g). Antioxidant assays, including TAC, FRAP, and DPPH, revealed significant antioxidant activity. Additionally, the aqueous extract exhibited notable antimicrobial efficacy, demonstrating minimum inhibitory concentrations (MICs) of 150 μg/mL against *Acinetobacter baumannii* and *Shigella* sp., and 2,500 μg/mL against *Aspergillus niger* and particular *Candida* species. The anticoagulant activity was evidenced by prolonged prothrombin time (98.9 s) and activated partial thromboplastin time (134.2 s at 11.5 mg/mL), and the antidiabetic activity was confirmed by the inhibition of α-amylase (EC_50_ = 104.8 μg/mL) and α-glucosidase (EC_50_ = 12.12 μg/mL) enzymes, as well as by a reduction in postprandial hyperglycemia in treated rats (400 mg/kg). *In silico* simulations revealed a strong affinity of pedunculagin for key biological targets, supporting its therapeutic potential. In conclusion, *Juglans regia* emerges as a promising natural resource for applications in the pharmaceutical, nutraceutical, and cosmetic fields, although clinical studies are required to validate its efficacy and safety.

## 1 Introduction

*Juglans regia* L., commonly known as the common walnut or Persian walnut, is a diploid tree species (2n = 32) belonging to the Juglandaceae family, a family distinguished by its great morphological and ecological diversity, comprising approximately 60 species distributed across 8 to 11 genera (Sharma et al., 2022). The genus *Juglans*, which includes 21 species, is particularly notable and is divided into four main sections based on fruit structure: *Trachycaryon*, *Cardiocaryon*, *Rhysocaryon*, and *Dioscaryon* (Manning, 1978). Among these, *Juglans regia* holds a central position due to its economic importance and wide geographical distribution. Several names, such as the Carpathian walnut, French walnut, or Himalayan walnut, are known for the species. Still, it is primarily called the common walnut or English walnut (Feng et al., 2018).

*Juglans regia* is a plant rich in bioactive compounds, particularly in its leaves, kernel, shell, and bark. The principal chemical constituents identified include flavonoids (e.g., quercetin and kaempferol), polyphenols (notably tannins and phenolic acids), juglones, sterols, triterpenes, and polyunsaturated fatty acids (particularly omega-3 and omega-6) (Ma et al., 2019; Hajipuor et al., 2022). These bioactive compounds are responsible for the plant’s diverse biological and pharmacological properties, contributing to its antioxidant, antimicrobial, anticoagulant, and antidiabetic activities. For instance, polyphenols and flavonoids are known for their potent antioxidant activities, while juglones, naphthoquinone compounds, exhibit antimicrobial and antifungal properties (Ahmad and Suzuki, 2019; Masek et al., 2019; Bennacer et al., 2022).

The biological properties of *J. regia* are extensive and diverse. Extracts derived from the leaves, husks, and walnut shells of *J. regia* have exhibited a broad spectrum of biological activities, including antioxidant, antimicrobial, anticoagulant, antidiabetic, anti-inflammatory, and anticancer properties (Jaiswal and Tailang, 2017; Al-Snafi, 2018). The antioxidants present in *J. regia* play a crucial role in neutralizing free radicals, thereby mitigating oxidative stress and preventing cellular damage associated with various chronic diseases and aging processes. The antimicrobial properties are particularly notable against pathogens such as *Escherichia coli*, *Staphylococcus aureus*, and *Candida albicans* (Vieira et al., 2019). Additionally, the bioactive compounds in *J. regia* have shown beneficial effects on cardiovascular health by reducing LDL cholesterol and improving endothelial function (Vieira et al., 2019). Pharmacological studies have also highlighted hypoglycemic impact, making it a promising candidate for diabetes management (Gupta et al., 2019).

*Juglans regia* has a long history of traditional use in various cultures worldwide. Traditional Chinese medicine uses walnut tree leaves and bark to treat skin infections, digestive disorders, and inflammatory diseases (Sharma et al., 2022). In India, walnuts are consumed to enhance cognitive health and prevent cardiovascular diseases, while the leaves are used in decoctions for their antiparasitic and antifungal properties (Bhardwaj et al., 2023). In Mediterranean countries, particularly Morocco, Greece, and Italy, walnuts are traditionally valued for their nutritional and medicinal benefits, especially for boosting the immune system and treating respiratory conditions (Tadayyon, 2013; Zaroual et al., 2019; Mukarram et al., 2024). In Morocco, walnut leaves are also used in infusions for their hypoglycemic and antihypertensive properties (Bourais et al., 2023).

*Juglans regia* cultivation in the Maghreb dates back to the Roman era when it was introduced and cultivated as a traditional fruit tree (Germain, 1997). Today, in Morocco, walnut trees are grown across an area of 7,600 ha, spanning diverse regions ranging from the humid and warm areas of the Rif and Atlas Mountains (High and Middle Atlas) to the arid areas in the southeast of the country (Kodad and Sindic, 2014). More than half of the walnut plantations in Morocco are propagated by seeds, a traditional method widely used by local farmers, while grafting remains a less common practice (Lansari et al., 2001). This form of cultivation has helped maintain significant genetic diversity, a hallmark of traditional Moroccan agroecosystems, where walnut trees coexist with other fruit species, such as olive and fig trees, sharing similar propagation methods and high levels of genetic diversity (Charafi et al., 2008; Khadari et al., 2008; Hssaini et al., 2020).

Morocco, located in an area of exceptional biodiversity within the Mediterranean basin, is home to remarkable genetic richness, particularly for tree species such as the walnut (Tous and Ferguson, 1996). This biodiversity is conserved mainly in traditional agroecosystems, where sustainable agricultural practices enable the management of plant and genetic resources differently from the intensive breeding programs often seen for other crops. However, this genetic diversity has not been sufficiently explored or leveraged for commercial and nutraceutical purposes despite numerous studies already highlighting the exceptional properties of *J. regia*.

The nutraceutical potential of *J. regia* has garnered increasing interest in recent years due to its bioactive properties and health benefits. Its leaves, kernel, shell, and bark contain a wide variety of bioactive compounds, including flavonoids, polyphenols, tannins, and phenolic acids, which are recognized for their antioxidant, antimicrobial, anticoagulant, and antidiabetic activities (Luna-Guevara et al., 2018; Verma and Sharma, 2020). Additionally, the by-products generated during walnut processing, such as shells and husks, which are often considered waste, are rich in phenolic compounds and represent an underutilized resource with significant nutraceutical potential. These parts of the plant could open new avenues for applications in various industrial sectors, including food, cosmetics, and pharmaceuticals.

Despite the growing interest in its benefits, the nutraceutical potential of *J. regia* cultivated in the Taza region of Morocco remains largely unexplored. This study aims to address this gap by analyzing the chemical profile and biological properties of *J. regia* from the Taza region, with a particular focus on its antioxidant, antimicrobial, anticoagulant, and antidiabetic activities. Through an integrated approach combining *in vitro*, *in vivo*, and *in silico* analyses, this research seeks to understand this species’s nutraceutical potential better and highlight its possible applications in the food and pharmaceutical industries. By examining bioactive compounds and their potential health effects, this study not only contributes to enriching scientific knowledge about the properties of *J. regia* but also underscores the importance of valorizing underutilized plant resources. This enhanced understanding could have significant implications for the sustainable development of agroecosystems while offering new economic opportunities for the Taza region and beyond.

## 2 Materials and methods

### 2.1 Vegetal material

**FIGURE 1:**
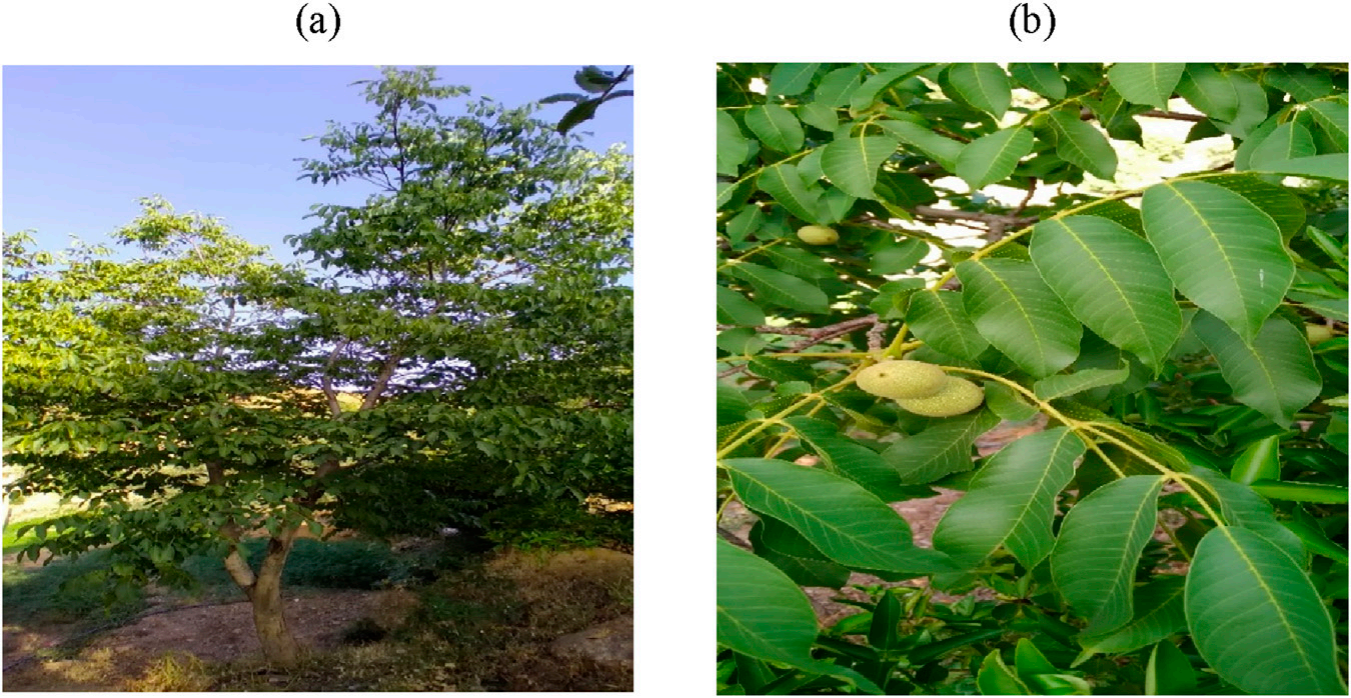
*Juglans regia* L.; **(a)**: Tree, **(b)**: Leaves and fruit.

The sample of *J. regia* analyzed was collected from cultivated stands in the Taza region (Figure 1). Table 1 provides detailed information regarding the origin, collected plant parts, and harvesting site. The plant species were formally identified at the Laboratory of Botany and Plant Ecology of the Scientific Institute of Rabat.

**TABLE 1:** Origin, part used, location, and harvesting period of *Juglans regia*.

Scientific name	Part Collected	Type of extract used	Harvesting area						
			Region	Province	Municipality	Latitude (x)	Longitude (y)	Altitude (m)	Collection period
*Juglans regia* L	husks	extract	Fez-Meknes	Taza	Taza	34° 11′11″N	4° 00′55″W	700 m	September 2024

### 2.2 Microbial materials

The antimicrobial activity of the aqueous extract of *J. regia* was evaluated against twenty-nine bacterial strains and eight fungal strains (Table 2). These pathogenic microorganisms are recognized for their high resistance, invasive potential, and human toxicity. They are frequently implicated in various infections in Morocco, posing significant clinical and therapeutic challenges. The tested strains were isolated from the hospital environment at Mohamed V Provincial Hospital in Meknes. All microbial strains were preserved in a 20% glycerol stock at −80°C, then revived in Mueller-Hinton and Sabouraud broths, and subcultured before experimentation to ensure viability and reproducibility of results.

**TABLE 2:** List of bacterial and fungal strains tested with their references.

Strains		Abbreviations	References
Gram-positive cocci	*Staphyloccocus epidermidis*	*S. epidermidis*	*5994*
	*Staphyloccocus aureus BLACT*	*S. aureus 2,510*	*4IH2510*
	*Staphyloccocus aureus STAIML/MRS/mecA/HLMUP/BLACT*	*S. aureus 2,220*	*2DT2220*
	*Streptococcus acidominimus*	*S. acidominimus*	*7DT2108*
	*Streptococcus group D*	*S. group D*	*3EU9286*
	*Streptococcus agalactiae*	*S. agalactiae*	*7DT1887*
	*Streptococcus porcinus*	*S. porcinus*	*2EU9285*
	*Enterococcus faecalis*	*E. faecalis*	*2CQ9355*
	*Enterococcuss faecium*	*E. faecium*	*13EU7181*
Gram-negative bacilli	*Acinetobacter baumannii*	*A. baumannii 2,404*	*7DT2404*
	*Acinetobacter baumannii*	*A. baumannii 2,410*	*7DT2410*
	*Escherichia coli*	*E. coli*	*3DT1938*
	*Escherichia coli ESBL*	*E. coli ESBL 2057*	*2DT2057*
	*Escherichia coli ESBL*	*E. coli ESBL 5765*	*2DT5765*
	*Enterobacter aerogenes*	*E. aerogenes*	*07CQ164*
	*Enterobacter cloacae*	*E. cloacae 317*	*02EV317*
	*Enterobacter cloacae*	*E. cloacae 2,280*	*2DT2280*
	*Citrobacter koseri*	*C. koseri*	*3DT2151*
	*Klebsiella pneumoniae* ssp. *pneumoniae*	*K. pneumoniae 1823*	*3DT1823*
	*Klebsiella pneumoniae* ssp. *pneumoniae*	*K. pneumoniae 1015*	*3DT1015*
	*Proteus mirabilis*	*P. mirabilis*	*2DS5461*
	*Pseudomonas aerogenosa*	*P. aerogenosa 2,138*	*2DT2138*
	*Pseudomonas aerogenosa*	*P. aerogenosa 1124*	*2DT1124*
	*Pseudomonas fluorescence*	*P. fluorescence*	*5442*
	*Pseudomonas putida*	*P. putida*	*2DT2140*
	*Serratia marcescens*	*S. marcescens*	*375BR6*
	*Salmonella* sp.	*Salmonella* sp.	*2CG5132*
	*Shigella* sp.	*Shigella* sp.	*7DS1513*
	*Yersinia enterocolitica*	*Y. enterocolitica*	*ATCC27729*
Yeasts	*Candida albicans*	*C. albicans*	*Ca*
	*Candida kefyr*	*C. kefyr*	*Cky*
	*Candida krusei*	*C. krusei*	*Ckr*
	*Candida parapsilosis*	*C. parapsilosis*	*Cpa*
	*Candida tropicalis*	*C. tropicalis*	*Ct*
	*Candida dubliniensis*	*C. dubliniensis*	*Cd*
	*Saccharomyces cerevisiae*	*S. cerevisiae*	*Sacc*
Fungi	*Aspergillus niger*	*A. niger*	*AspN*

### 2.3 Animal selection for research

The acute toxicity study was conducted on albino mice (both male and female), while the *in vivo* antidiabetic activity assessment was performed using Wistar rats (both male and female). All animals were housed under controlled environmental conditions, maintaining a 12-h light/dark photoperiod and a temperature of 22°C ± 2 °C in the animal facility of the Biology Department at the Faculty of Sciences Dhar El Mehraz. Throughout the experimental procedures, strict adherence to ethical guidelines and regulations was ensured to safeguard animal welfare. The study was reviewed and approved by the Institutional Ethics Committee for the Care and Use of Laboratory Animals at the Faculty of Sciences Dhar El Mehraz, Sidi Mohamed Ben Abdallah University, Fez, Morocco (04/2019/LBEAS) (Albus, 2012).

### 2.4 Quality control of plant material

#### 2.4.1 Moisture content

The moisture content was determined following the AFNOR standard (NF-V03-402, 1985) (NF V03-402, 1985). A 5 g portion of the plant sample was precisely weighed into pre-dried and tared crucibles to ensure accurate measurement. The crucibles containing the plant material were placed in an oven at 103°C–105°C for 24 h. After this period, the crucibles were cooled in a desiccator before being weighed again. The moisture content was calculated using the formula provided below (Formula 1):

$$MC\%=\left(\frac{m_{0}-m_{1}}{m_{0}}\right)\times 100$$ (1)

With: m_0_: initial mass of the plant in (g); m_1_: mass after drying in (g).

The result is expressed as a percentage of dry matter.

#### 2.4.2 Determination of pH

The product’s acidity is assessed through its pH measurement. The method involves mixing 2.0 g of the sample with 10 mL of warm distilled water. After filtration, the mixture is cooled, and the pH is measured by immersing the electrode in an adequate amount of the filtrate (Saidi et al., 2023).

#### 2.4.3 Ash content

The ash content represents the mineral residue that remains after the combustion of organic matter at high temperatures in a muffle furnace. To determine this, a 5 g portion of the ground plant sample was incinerated at 550°C until all carbonized particles were eliminated, yielding a stable weight of whitish ash per the NF ISO 5984 standard. Subsequently, the organic matter content was calculated using the following Formula 2:

$$OM\%=\left(\frac{m_{1}-m_{2}}{\text{TE}}\right)\times 100$$ (2)

OM%: Organic Matter

m_1_: Weight of the capsule and sample before calcination

m_2_: Weight of the capsule and sample after calcination.

TE: Test portion.

The ash content was calculated as follows (Formula 3):

Ash %=100−OM% (3)

#### 2.4.4 Heavy metal analysis: inductively coupled plasma atomic emission spectrometry (ICP-AES)

Several heavy metals, including arsenic (As), cadmium (Cd), chromium (Cr), iron (Fe), lead (Pb), antimony (Sb), and titanium (Ti), were investigated. Each of these metals has specific contamination limits (Makarem et al., 2024). Exceptions apply to medicines whose main ingredients are known to accumulate significant levels of cadmium naturally. To analyze the concentrations of these elements, the mineralization protocol outlined in the AFNOR standard (1999) was employed, using aqua regia (HNO_3_ + 3 HCl). This approach ensures the preparation of sufficiently large samples, reducing representativity issues. The procedure involved combining 0.1 g of finely ground plant material with 3 mL of aqua regia, made up of 1 mL of concentrated nitric acid (HNO_3_; 99%) and 2 mL of hydrochloric acid (HCl; 37%). The mixture was heated under reflux at 200 °C for 2 hours, then cooled and left to settle. The supernatant was extracted, filtered through a 0.45 µm membrane, and diluted to a final volume of 15 mL using distilled water. The concentrations of heavy metals were determined through ICP-AES analysis (Ultima 2 Jobin Yvon) at the UATRS laboratory (Technical Support Unit for Scientific Research) of the CNRST in Rabat (Skujins, 1998). It is worth noting that ICP-based analytical techniques are widely used in pharmaceutical researches (Klika et al., 2024).

#### 2.4.5 Phytochemical screening

This qualitative study aimed to identify chemical families through solubility, precipitation, and turbidity tests. Additional techniques included observing specific color changes and examining samples under UV light. The research focused on the husks of *J. regia* for phytochemical analysis. Dried plant material was finely ground into powder, and the characterization of various chemical groups was carried out following the methodologies outlined by Dohou et al. (2003), Judith. (2005), Bekro et al. (2007), Mezzoug et al. (2007), Bruneton (2009), N'Guessan et al. (2009).

### 2.5 Study of phenolic compounds

#### 2.5.1 Extraction of phenolic compounds

In pharmaceutical and medicinal chemistry, phenol-based compounds are considered as multifunctional agents and are widely used in drug discovery (Rappoport, 2004; Klika et al., 2022; Makarem et al., 2025). Therefore, it is important to identify and investigate them in medicinal plant extract. Phenolic compounds were extracted using two complementary techniques, decoction and solid-liquid extraction via a Soxhlet apparatus, to optimize the recovery of bioactive molecules. In the decoction method, 30 g of plant material was immersed in 600 mL of distilled water, heated to 80 °C, and maintained at this temperature for 1 h to facilitate the release of phenolic compounds. The mixture rested for 5 minutes, followed by filtration under reduced pressure to remove insoluble residues. The resulting extract was subsequently dried in an oven at 70 °C, collected as a powder in a glass vial, and stored for further analysis. In parallel, the Soxhlet extraction method was applied to two additional 30 g samples, utilizing 300 mL of either distilled water or a hydroethanolic solution (70:30, v/v) as the extraction solvent. Following multiple extraction cycles, the obtained extracts were concentrated using a rotary evaporator to remove excess solvent and enhance compound purity. A detailed codification of the extracts prepared in this study is presented in Table 3.

**TABLE 3:** Extraction coding.

Extraction methods	Solvents	Codification
Soxhlet	Ethanol/Water (70/30; *v*/*v*)	E (2)
	Water	E (1)
Decoction	Water	E (0)

#### 2.5.2 Determination of total polyphenols

The total polyphenol content in the studied plant extracts was determined using the Folin-Ciocalteu method, following the protocol established by Singleton and Rossi (Singleton and Rossi, 1965). This widely recognized technique is based on the oxidation of polyphenolic compounds, leading to a characteristic blue coloration. The method involves the reduction of the Folin-Ciocalteu reagent, a mixture of phosphotungstic acid (H_3_PW_12_O_40_) and phosphomolybdic acid (H_3_PMo_12_O_40_), into their respective blue oxides (W_8_O_23_ for tungsten and Mo_8_O_3_ for molybdenum). The quantification was carried out colorimetrically, where the optical density of the solution was measured at 760 nm using a UV-Vis spectrophotometer (UV mini-1240). A blank sample (reaction mixture without extract) was used as a reference to ensure accuracy. Gallic acid was employed as the positive control, prepared at a 50 μg/mL concentration, and a calibration curve was generated under identical experimental conditions. The results were expressed as milligrams of gallic acid equivalent per Gram of extract (mg GAE/g), calculated using formula Y = ax + b, derived from the calibration curve. To ensure the reliability of the measurements, each analysis was conducted in triplicate.

#### 2.5.3 Determination of flavonoids

The flavonoid content in the studied plant extracts was determined using the aluminum chloride (AlCl_3_) colorimetric method, adapted from the protocols established by Djeridane (Djeridane et al., 2006) and Hung (Hung et al., 2009), along with their collaborators. This method is based on the reaction between aluminum chloride and the hydroxyl (–OH) groups of flavonoids, forming a stable flavonoid-AlCl_3_ complex, which exhibits characteristic absorbance. Quantification was carried out using UV spectroscopy at a wavelength of 433 nm. Quercetin, a well-characterized flavonoid, was employed as the reference standard and subjected to the same analytical conditions as the plant extracts. A calibration curve was established following formula Y = ax + b, with quercetin concentrations ranging from 5 to 30 μg/mL. The flavonoid content was subsequently expressed as milligrams of quercetin equivalent per Gram of extract (mg QE/g). All experiments were performed in triplicate to ensure the accuracy and reproducibility of the measurements.

#### 2.5.4 Determination of condensed tannins

The condensed tannin content in the studied plant extracts was estimated using the vanillin method (Price et al., 1978), a widely applied technique for tannin quantification. Briefly, various concentrations of a prepared (+)-catechin solution (2 mg/mL) were mixed with 3 mL of a 4% (m/v) vanillin/methanol solution, followed by manual agitation to ensure homogeneity. Subsequently, 1.5 mL of concentrated hydrochloric acid was added to each concentration, and the reaction mixtures were allowed to incubate for 20 min at room temperature to facilitate color development. Absorbance measurements were recorded at 499 nm using a UV-Vis spectrophotometer, with a blank sample as a reference. The tannin content in the plant extracts was determined by substituting catechin with the sample extracts, following the same calibration curve process. The final tannin concentration was expressed as milligrams of catechin equivalent per Gram of dry weight material (mg CE/g DW).

#### 2.5.5 Determination of hydrolyzable tannins

The hydrolyzable tannin content was determined using the method of Willis and Allen (Willis, 1998), with slight modifications to optimize accuracy. In this procedure, 10 µL of the plant extract was vortexed for 10 s with 5 mL of a 2.5% potassium iodate (KIO_3_) solution, ensuring thorough mixing. The maximum absorbance was reached after 2 min for the plant extract (optimal reaction time) and after 4 min for the tannic acid standard solution, indicating different reaction kinetics. Absorbance was subsequently recorded at 550 nm using a UV-Vis spectrophotometer. The results were expressed as milligrams of tannic acid per Gram of dry plant material (mg TAE/g). A calibration curve was established using 11 different tannic acid concentrations ranging from 100 to 2000 μg/mL to ensure precise quantification.

#### 2.5.6 HPLC/UV ESI-MS analysis of *Juglans regia* extracts

The phenolic composition of the *J. regia* decoction was analyzed using high-performance liquid chromatography (HPLC) coupled with Q Exactive Plus mass spectrometry and electrospray ionization (HPLC/UV-ESI-MS), ensuring a highly sensitive and accurate identification of bioactive compounds. The analysis was performed on an UltiMate 3000 HPLC system (Thermo Fisher Scientific) with a reverse-phase C18 column (250 × 4 mm, 5 μm, Merck), maintained at 40 °C. At the same time, the samples were stored at 5 °C before injection. The mobile phase consisted of solvent A (0.1% formic acid in water) and solvent B (0.1% formic acid in acetonitrile), following a gradient elution from 2% to 95% of solvent B over a 30-min runtime, ensuring optimal separation of phenolic constituents. The flow rate was 1 mL/min, and the injection volume was 20 μL.

Detection was carried out using mass spectrometry in MS/MS mode (bbCID) on a Maxis Impact HD mass spectrometer (Bruker Daltonik) operating under negative ionization mode. Key operating parameters included a capillary voltage of 3000 V, a drying gas temperature of 200 °C, a dry gas flow rate of 8 L/min, a nebulizer gas pressure of 2 bar, and a plate offset of −500 V, with nitrogen employed as the desolvation and nebulization gas.

Additionally, UV detection was performed using a diode array detector (Merck-Hitachi), scanning wavelengths in the range of 190–600 nm, with specific acquisitions at 280 nm, 320 nm, and 360 nm, targeting different classes of phenolic compounds. Mass spectrometric data were acquired over an *m/z* range of 100–1500 and subsequently processed using Chromeleon™ 7.2 software (Thermo Scientific), facilitating compound identification based on the mass spectra of eluted molecules.

### 2.6 Antioxidant activities

#### 2.6.1 Antiradical activity by the DPPH• test

The ability of antioxidant chemicals, especially phenolics, to give up a single electron and stabilize the synthetic DPPH∙ radical (2,2-diphenyl-1-picrylhydrazyl) was used to measure antiradical activity. This reaction causes a colorimetric shift from violet to yellow, which can be measured in numbers. The test was done with a UV-visible spectrophotometer that had a wavelength of 515 nm (Brand-Williams et al., 1995). To make the DPPH∙ solution, 2.4 mg of DPPH∙ was mixed with 100 mL of ethanol. This made a 6 × 10^−5^ M solution. Ethanol was also used to dissolve the extract samples so that they would all be the same. In the experiment, 200 µL of the plant extract (sample) or a standard antioxidant (ascorbic acid) at different doses were mixed with 2.8 mL of the DPPH∙ solution that had already been made. The mixes were then left in the dark at room temperature for 30 min to keep the radical species from breaking down from light. After that, absorbance was measured at 515 nm, using a blank sample that simply had ethanol in it as a reference. We utilized a DPPH solution without extract as the negative control to set the baseline absorbance values. Using Formula 2 (Tagnaout et al., 2016), the findings were shown as a percentage of inhibition. This made it possible to compare the antioxidant potential of the plant extracts to the reference standard.

$$\%\text{ AA}=\frac{\text{Abs}\;\text{control}-\text{Abs}\;\text{sample}}{\text{Abs}\;\text{control}}\times 100$$ (4)

Where: % AA: percentage of antiradical activity; Abs control: absorbance of the blank (optical density of the solution consisting of DPPH^·^ and ethanol); Abs sample: absorbance of the test compound (extracts).

#### 2.6.2 Ferric reducing antioxidant power method (FRAP)

We used the Ferric Reducing Antioxidant Power (FRAP) test, which is based on the Oyaizu (1986) method, to see how well the plant extracts could lower iron levels. This method depends on changing Fe^3+^ in the K_3_Fe(CN)_6_ complex to Fe^2+^, which shows how well an antioxidant works. The experimental approach used the same treatment settings as the prior samples, which made sure that the analysis was consistent. We used a UV-Vis spectrophotometer to measure absorbance at 700 nm to find out how much reducing power there was. We used distilled water to calibrate the instrument. A blank sample was made in the same way as the other samples to use as a reference. We also utilized a standard antioxidant solution (ascorbic acid) as a positive control and assessed its absorbance at the same time as the plant extracts. The results showed that an increase in absorbance was linked to an increase in reducing power, which means that the examined extracts had a stronger antioxidant ability (Oyaizu, 1986; Končić et al., 2010).

#### 2.6.3 Total antioxidant capacity (TAC)

We used the phosphomolybdenum method, as Khiya et al. (2021) describes, to find out the plant extracts’ Total Antioxidant Capacity (TAC). This method works by changing molybdenum (VI), which is found as molybdate ions (MoO_4_^2-^), into molybdenum (V) (MoO_2_^+^) in the presence of antioxidant chemicals. This causes a green phosphate/Mo(V) complex to form in acidic circumstances. The reagent solution for the experiment was made up of 0.6 M sulfuric acid, 28 mM sodium phosphate, and 4 mM ammonium molybdate. It was combined with 0.3 mL of the extract. The reaction mixtures were then sealed and put in an incubator at 95 °C for 90 min to make sure the reduction was complete. We used a UV-Vis spectrophotometer to measure the absorbance of the solutions after they had cooled to room temperature. A blank sample made in the same way served as the reference. To find out how much antioxidant power there was, a standard calibration curve was made using different amounts of ascorbic acid. The results were shown as milligrams of ascorbic acid equivalents per Gram of crude extract (mg AAE/g CE). This gave a way to compare the overall antioxidant capability of the extracts.

### 2.7 Determination of the minimum inhibitory concentration, minimum bactericidal concentration, and minimum fungicidal concentration

We used 96-well microplates and the reference microdilution method (Balouiri et al., 2016) to find the Minimum Inhibitory Concentration (MIC). MIC is the smallest amount of decoction extract that can stop the tested microorganism from growing during incubation. We made a series of dilutions using a stock solution of the decoction extract dissolved in 10% DMSO. The quantities of each decoction extract ranged from five to 0.93 × 10^−2^ mg/mL. We made these dilutions in a final volume of 100 µL using Sabouraud broth for fungus and Mueller-Hinton medium for bacteria. After that, 100 µL of microbial inoculum was added to each dilution step. The final concentration was 10^6^ CFU/mL for bacteria and 10^4^ CFU/mL for fungi. To check how much bacteria had grown after being incubated at 37 °C for 24 h, 10 μL of resazurin were added to each well. The fact that the hue changed from purple to pink after being incubated at 37 °C for 2 hours showed that microbes were growing. The MIC value was found to be the lowest concentration that kept re-sazurin from changing color. The 11th and 12th wells had controls for growth and sterility, respectively. The decoction extract was tested twice. We mashed up 250 mg of terbinafine, a common antifungal drug utilized in the study, and mixed it with 2 mL of 10% DMSO for comparison. We found the Minimum Bactericidal Concentration (MBC) and Minimum Fungicidal Concentration (MFC) by taking 10 µL from wells with no apparent growth and putting it on Mueller-Hinton agar (for bacteria) or Sabouraud broth (for fungus) and letting it sit at 37 °C for 24 h. The MBC or MFC was the lowest amount of the sample that caused a 99.99% drop in CFU/mL relative to the control. We also figured out the MBC/MIC or MFC/MIC ratio for each extract to see how well it worked against germs. If the ratio was less than 4, the decoction extract was thought to kill bacteria and fungi; if it was more than 4, it was thought to stop bacteria and fungi from growing (Bissel et al., 2014). The biological assays were conducted in accordance with globally recognized standardized protocols, following the guidelines of the CLSI (Clinical and Laboratory Standards Institute) for antimicrobial testing.

### 2.8 Anticoagulant activity

We tested the anticoagulant capabilities of the *J. regia* decoctions we made by using time-based coagulation tests, especially Prothrombin Time (PT) and Activated Partial Thromboplastin Time (aPTT), following the method published by Hmidani et al. (2019). We looked at extract concentrations between 11.500 mg/mL to 0.179 mg/mL to see if they would have anticoagulant effects. To prepare plasma, blood samples were put in tubes with 3.8% trisodium citrate and then spun at 25,000 rpm for 10 min to separate the platelet-poor plasma. The plasma was then combined and kept at −10 °C until it could be looked at again. We combined 50 µL of pooled citrated plasma with 50 µL of the plant extract solution and let it sit at 37 °C for 10 min in the aPTT test. After that, 100 µL of PTT reagent (CKPREST^®^, Diagnostica Stago) was added, and the mixture was kept at 37 °C for another 5 min. After that, 100 µL of 25 mmol/L calcium chloride (CaCl_2_) was added to start coagulation, and the time it took for the blood to clot was noted. To do the PT test, 50 µL of pooled citrated plasma was combined with 50 µL of the plant extract solution and then left to sit for 10 min. After that, 200 µL of Neoplastin^®^ Cl reagent was added, and the mixture was preincubated at 37 °C for additional 10 min before the clotting time was measured. The aqueous extracts’ ability to stop blood from clotting was measured in seconds at different concentrations. We used an automated coagulometer (MC4Plus, MER-LIN Medical^®^) to do each measurement six times to make sure they were accurate and could be repeated.

### 2.9 Antidiabetic activity

#### 2.9.1 Study of the inhibitory effect of aqueous extracts on the activity of pancreatic α-amylase, *in vitro*

We tested the aqueous extract’s ability to stop α-amylase using the method set out by Daoudi et al. (Daoudi et al., 2020), with acarbose as a positive control. The test was done with different amounts of acarbose (1, 0.8, 0.6, 0.4, and 0.2 mg/mL) and the aqueous extract (0.89, 0.45, 0.22, 0.11, and 0.06 mg/mL). For the test, 200 µL of the acarbose solution or the aqueous extract solution was mixed with a phosphate buffer solution to make sure the enzymes stayed stable. To make a blank sample, the enzyme solution was replaced with 200 µL of phosphate buffer. This made it possible to adjust for background noise. Before the experiment, all of the reaction tubes were kept at 37 °C for 10 min. Then, 200 µL of starch solution was added to each tube as the enzyme substrate. The reaction mixtures were then kept at 37 °C for 15 min to make sure that the enzyme and substrate fully interacted. To stop the enzymes from working, 600 µL of the 3,5-dinitrosalicylic acid (DNSA) reagent was added. Then, the mixture was put in boiling water for 8 min to let the colorimetric reaction happen. After being heated, the reaction quickly stopped because of thermal shock, and the tubes were then cooled in an ice bath. Before measuring absorbance at 540 nm with a UV-Vis spectrophotometer, 1 mL of distilled water was added to each tube to make sure the sample contents were the same. The blank sample, which just had the buffer solution and no enzyme, was used as a reference to fix the background absorbance. Using Formula 3, we figured out the percentage of α-amylase inhibition for each extract or acarbose concentration. This gave us an idea of how the extract would work against diabetes by stopping the breakdown of carbohydrates.

$$\%\text{ Inhibition}=\frac{A\;\text{control}-A\;\text{sample}}{A\;\text{control}}\times 100$$ (5)

Where: A control: absorbance of enzyme activity without inhibitor; A sample: absorbance of enzymatic activity in the presence of extract or acarbose.

#### 2.9.2 Study of the inhibitory effect of aqueous extracts on the activity of α-glucosidase, *in vitro*

Using p-nitrophenyl-α-D-glucopyranoside (pNPG) as the substrate, we tested the *J. regia* extract’s ability to block α-glucosidase according to the method reported by Chatsumpun et al. (2017), with some changes. The extracts were evaluated at doses between 0.488 and 100 μg/mL to get a full picture of their possible antidiabetic effects. We made each sample in 5% dimethyl sulfoxide (DMSO), which was also utilized as the solvent control. To keep the enzymatic conditions at their best, we dissolved the α-glucosidase enzyme in phosphate buffer (pH 6.8). We employed acarbose, a well-known α-glucosidase inhibitor, as the positive control. The test was done in a 96-well microplate, where 40 µL of α-glucosidase enzyme (0.1 U/mL) and 10 µL of each extract sample were introduced to the wells one after the other. The mixture was put together and kept at 37 °C for 10 min to let the enzymes and substrates interact for the first time. Next, 50 µL of pNPG (1 mM) was added as the substrate for the enzyme, and the reaction mixture was kept at 37 °C for another 20 min to make sure that the hydrolysis was complete. To stop the reaction, 100 µL of sodium carbonate (Na_2_CO_3_, 0.1 M) was added to each well. This raised the pH, which stopped the enzymes from working. We used a microplate reader to measure absorbance at 405 nm to find out how much of the reaction product, p-nitrophenol, there was. Formula 3 was used to figure out the percentage of α-glucosidase that was blocked. This made it possible to compare the effectiveness of the extract with that of acarbose and see if it might be used as a natural antidiabetic drug.

#### 2.9.3 Acute toxicity study

The objective of this study was to find out how safe the therapeutic dose of *J. regia* extract was in the short term by testing its acute oral toxicity in normal mice, which is a common way for people to be exposed to it. The OECD rules (Weng et al., 2021) were followed during the investigation to make sure that international safety standards were met. We gave the aqueous extract (E0) in three different amounts: 0.5, 1, and 2 g/kg. Before the experiment, albino mice that weighed 20–35 g were not fed for 14 h. Then, they were randomly put into four groups (n = 6; ♂/♀ = 1). The control group got 10 mL/kg of distilled water, while the three test groups got E0 at the levels shown. After giving the mice a single dose by mouth, they were closely watched for 10 h to look for any indicators of toxicity, such as changes in behavior, physical distress, or bad reactions. Also, daily observations were made for 14 days to look for any behavioral or clinical problems that might show up later and could be signs of possible harmful effects. This methodical methodology made sure that the safety profile of the extract at the tested levels was thoroughly evaluated.

#### 2.9.4 Study of the antihyperglycemic activity of the aqueous extract of *Juglans regia* in normal rats *in vivo*

We did the oral glucose tolerance test (OGTT) and the oral sucrose tolerance test (OSTT) on rats that were already normal in blood sugar levels and then gave them a lot of *D*-glucose to see how well the aqueous extract (E0) could lower their blood sugar levels after eating. This is important for managing metabolic disorders like diabetes. To do this, 200–250 g rats that had been fasting for 14 h were randomly put into three experimental groups (n = 6; ♂/♀ = 1). One group got distilled water (10 mL/kg), another group got E0 (2 mL/kg), and a third group got glibenclamide (2 mg/kg), a common hypoglycemic drug. Before the therapy, the rats were put to sleep with ether, and their blood sugar levels were measured to set a baseline (t_0_). After giving the test substances (E0, distilled water, or glibenclamide) by mouth, blood glucose levels were checked again after 30 min. Then, a *D*-glucose overload (2 mg/kg) was given to cause a postprandial glycemic response. After that, blood sugar levels were checked every 30, 60, 90, and 150 min for 3 hours. This made it possible to compare the glycemic response between the different treatment groups. This experimental method gave us useful information about the extract’s ability to lower blood sugar and its ability to change how glucose is processed when carbohydrates are eaten.

### 2.10 Molecular docking

The three-dimensional structures of the protein targets listed in Table 4 were retrieved from the RCSB Protein Data Bank (https://www.rcsb.org/) and visualized using UCSF Chimera to facilitate molecular docking analysis. Protein structures were preprocessed using Chimera and AutoDock tools (version 1.5.6, The Scripps Research Institute, La Jolla, CA, United States), which involved the removal of water molecules, heteroatoms, unwanted protein chains, and co-crystallized ligands to optimize docking conditions. To ensure accurate molecular interactions, polar hydrogens were added, and Gasteiger charges were assigned before converting the protein structures into pdbqt format for docking simulations. The three-dimensional models of the seven studied compounds were retrieved from PubChem, followed by energy minimization to ensure structural stability. Ligands were then processed using OpenBabel to convert SDF files into pdbqt format, ensuring compatibility with docking simulations. AutoDock Vina was employed to conduct molecular docking, utilizing a scoring function to evaluate binding affinities. A grid was systematically defined over the protein structure, precisely targeting the active site residues, and its dimensions and coordinates were carefully optimized to align with the binding pocket before initiating the docking procedure. Post-docking analysis was performed using PyMOL, where ligand-protein interactions were examined, and binding affinities were expressed in terms of free energy values. To validate the accuracy of the docking protocol, the co-crystallized ligand was removed, and re-docking was performed within the same binding region to confirm the reliability of the methodology. The overlap between the docked ligand and the original crystallized ligand was quantitatively assessed using the root-mean-square deviation (RMSD) value, ensuring that the docking results were statistically and structurally valid.

**TABLE 4:** Protein targets and molecular docking parameters.

Activities	Targets	PDB ID	Grid box center coordinates	Grid box size
Antibacterial activity	Isoleucyl-tRNA synthetase	1JZQ	center_x = −27.803 center_y = 6.619 center_z = −28.722	size_x = 34 size_y = 21 size_z = 23
	DNA gyrase	1KZN	center_x = 18.325 center_y = 30.783 center_z = 36.762	size_x = 20 size_y = 38 size_z = 38
	Dihydropteroate synthase	2VEG	center_x = 31.404 center_y = 48.530 center_z = 0.204	size_x = 24 size_y = 24 size_z = 18
	Dihydrofolate Reductase in complex with NADPH and trimethoprim	2W9G	center_x = −4.067 center_y = −27.461 center_z = 4.035	size_x = 24 size_y = 28 size_z = 28
	D-Alanine Ligase	2ZDQ	center_x = 47.378 center_y = 12.782 center_z = 5.730	size_x = 23 size_y = 26 size_z = 32
	Dihydrofolate Reductase complexed with novel 7-aryl-2,4-diaminoquinazolines	3SRW	center_x = −4.701 center_y = −31.536 center_z = 6.341	size_x = 26 size_y = 28 size_z = 23
	Penicillin-binding protein 1a PBP1a	3UDI	center_x = 34.198 center_y = −1.249 center_z = 12.715	size_x = 24 size_y = 24 size_z = 28
	Crystal Structure of Staph ParE 24 kDa	4URN	center_x = −31.684 center_y = 8.021 center_z = −4.598	size_x = 28 size_y = 40 size_z = 38
	Carbapenem-hydrolyzing beta-lactamase KPC	4ZBE	center_x = 3.005 center_y = 2.027 center_z = 12.341	size_x = 26 size_y = 42 size_z = 34
	Oxygen-insensitive NAD(P)H nitroreductase	5J8G	center_x = 36.826 center_y = 45.915 center_z = −24.412	size_x = 23 size_y = 24 size_z = 22
	Anthranilate--CoA ligase	5OE3	center_x = 38.132 center_y = −3.329 center_z = 14.677	size_x = 32 size_y = 26 size_z = 20
	Catalase compound II	2CAG	center_x = 60.017 center_y = 14.760 center_z = 15.935	size_x = 28 size_y = 22 size_z = 34
	Acriflavine resistance protein B	4DX5	center_x = 40 0.005 center_y = −25.012 center_z = −32.117	size_x = 24 size_y = 26 size_z = 32
	Beta-lactamase NDM-1	4HL2	center_x = −4.007 center_y = −5.025 center_z = 16.291	size_x = 26 size_y = 34 size_z = 38
	Cell division protein FtsZ	3VOB	center_x = −7.391 center_y = 0.102 center_z = 16.068	size_x = 26 size_y = 34 size_z = 38
Antifungal activity	CYP51 VARIANT1	5FSA	center_x = 205.007 center_y = 14.197 center_z = 58.034	size_x = 28 size_y = 24 size_z = 32
	Lanosterol 14-alpha demethylase	5V5Z	center_x = −44.007 center_y = −14.161 center_z = 22.011	size_x = 24 size_y = 30 size_z = 36
Antioxidant activity	Lipoxygenase-3	1N8Q	center_x = 26.014 center_y = 0.014 center_z = 16.108	size_x = 22 size_y = 28 size_z = 32
	Cytochrome P450 2C9	1OG5	center_x = −38.207 center_y = 61.001 center_z = 27.024	size_x = 22 size_y = 28 size_z = 32
	NADPH oxidase	2CDU	center_x = 18.26 center_y = −6.350 center_z = −1.530	size_x = 24 size_y = 22 size_z = 28
	Xanthine dehydrogenase/oxidase	3NRZ	center_x = 58.097 center_y = 3.009 center_z = 35.108	size_x = 20 size_y = 28 size_z = 34
	Superoxide Dismutase	1HL5	center_x = 27.097 center_y = 111.039 center_z = 64.117	size_x = 22 size_y = 30 size_z = 32
	Glutathione peroxidase 1	2F8A	center_x = −8.001 center_y = 20.237 center_z = 19.842	size_x = 26 size_y = 28 size_z = 30
Antidiabetic activity	Pancreatic alpha-amylase	4W93	center_x = −9.004 center_y = 22.197 center_z = −17.361	size_x = 28 size_y = 22 size_z = 36
	Alpha-glucosidase	3W37	center_x = 16.169 center_y = −19.023 center_z = −31.110	size_x = 20 size_y = 22 size_z = 28
Anticoagulant activity	Thrombin Heavy Chain	4UFD	center_x = 13.087 center_y = −1.004 center_z = 18.0964	size_x = 24 size_y = 24 size_z = 30

### 2.11 Molecular dynamics simulation

We used GROMACS 2019.3 to do molecular dynamics (MD) simulations to test how stable and dynamic the protein-ligand interactions were (Van Der Spoel et al., 2005). We used the CHARMM36 all-atom force field to set the protein’s parameters and the CGenFF server to make ligand topologies that accurately depict the molecules. To keep the system neutral, counterions were added to balance out the net charges. Each protein-ligand combination was placed in a rectangular simulation box filled with TIP3P water molecules, which simulated real-life conditions. Before the simulation phase, the steepest descent approach was used to minimize energy. The highest force threshold (Fmax) was established at 1000 kJ/mol/nm to make sure the system’s structure stayed stable and there were no steric conflicts. The MD simulations were run in NVT (constant number of particles, volume, and temperature) and NPT (constant number of particles, pressure, and temperature) ensembles, which kept the thermodynamic stability over the whole process. We did a 100-nanosecond MD simulation for each system to see how flexible and stable the protein-ligand complexes would be over time. We carefully looked at the resulting trajectory files to find important values like root-mean-square deviation (RMSD), root-mean-square fluctuation (RMSF), radius of gyration (Rg), and hydrogen bonding interactions. This gave us molecular-level information about how stable the binding is and how it changes over time.

### 2.12 MM-PBSA binding energy calculation

The Molecular mechanics Poisson–Boltzmann surface area (MM-PBSA) was used to compute the binding free energies of the complexes (https://pubmed.ncbi.nlm.nih.gov/24850022/). The calculations were conducted using the g_mmpbsa script tool (Dasmahapatra et al., 2022), which employs an approach based on the average of two energy values: the solvation energy and the potential energy in a vacuum.

$$\Delta E\;\left(\text{MM}-\text{PBSA}\right)=\Delta \text{EMM}+\Delta \text{Gsolvation}$$ (6)

In Formula 4, EMM represents the potential energy in a vacuum, while Gsolvation corresponds to the free solvation energy. The molecular mechanical energy (EMM) is determined by considering the contributions of the electrostatic component (Eele) and the van der Waals interaction (EvdW). The solvation energy is calculated using the polar solvation energy (Gpol) and the non-polar solvation energy (Gnonpol). The polar solvation energy (Gpol) is determined using the Poisson-Boltzmann formula (PB), while the non-polar solvation energy (Gnonpol) is evaluated based on the solvent-accessible surface area (SASA).

### 2.13 Statistical analysis

The results were expressed as mean values ±standard error of the mean (SEM) to ensure accurate representation of data variability. Statistical analyses were performed using one-way analysis of variance (ANOVA), followed by Tukey’s *post hoc* test to determine significant differences between groups. All analyses were conducted using GraphPad Prism 9 (version 9.5.1, San Diego, CA, United States), a widely recognized statistical software for biological and pharmacological research. A significance threshold of *p < 0.05* was applied to establish statistical relevance, ensuring that only robust and meaningful differences were considered in the interpretation of the results. Correlations between phenolic compound content and antioxidant activities were examined using R software (version 4.4.2).

## 3 Results

### 3.1 Quality control of plant material

The results of the quality control of plant material, presented in Tables 5, 6, highlight the remarkable properties of *J. regia* husks, confirming its potential for nutraceutical and pharmacological applications. With a moisture content of 11.06%, which is below the 12% threshold recommended by the FAO, the material demonstrates excellent preservation capabilities. Its neutral pH (6.01) and high mineral content (17.41% ash) reflect optimal chemical stability and a rich nutritional profile. Furthermore, the analysis of heavy metals within the Juglans genus has been minimally studied. Seven elements (As, Cd, Cr, Fe, Pb, Sb, and Ti) were determined in our case. The concentrations strictly adhere to FAO/WHO standards, ensuring the product’s safety and security. These results underscore the potential of *J. regia* as a reliable and high-quality resource for applications in the health and nutrition fields.

**TABLE 5:** Moisture Content, pH, and Ash Content in *Juglans regia* husks.

Species	Moisture content (th%)	pH	Ash content (%)
*J. regia*	12.06 ± 0.60	6.01 ± 0.30	17.41 ± 0.87

**TABLE 6:** Heavy metal concentration (mg/L) and maximum limits FAO/WHO (2009).

Species	Arsenic (As)	Cadmium (Cd)	Chromium (Cr)	Iron (Fe)	Lead (Pb)	Antimony (Sb)	Titanium (Ti)
*J. regia*	0.1189	0.0443	0.0872	0.901	0.1008	0.1221	0.0758
Maximum limits (FAO/WHO)	1	0.3	2	20	3	1	-

### 3.2 Phytochemical tests on *Juglans regia* husks

Phytochemical analysis of the *J. regia* husks revealed the presence of several biologically significant secondary metabolites, as summarized in Table 7. Notably, a strongly positive reaction was observed for steroids, triterpenes, and saponins, indicating a high concentration of these bioactive compounds. Flavonoids and tannins, both catechic and gallic, were also detected, although their presence was classified as moderate. A weak positive reaction was noted for quinones and O-type and C-type heterosides regarding anthraquinone derivatives. In contrast, tests showed a complete absence of sugars, holosides, and alkaloids, as evidenced by the adverse reactions to Dragendorff and Mayer reagents. These results highlight the bioactive compound richness of *J. regia* husks, particularly in steroids, triterpenes, and saponins, which play a key role in this species’ biological and pharmacological properties. The notable presence of flavonoids and tannins further enhances these extracts’ antioxidant and antimicrobial potential. Conversely, the absence of alkaloids and simple sugars suggests a distinct chemical profile, which could guide future research toward exploring these specific compounds for nutraceutical or pharmaceutical applications.

**TABLE 7:** Results of phytochemical tests.

Compounds/Species		*J. regia*
Part used		Husks
Sterols and triterpenes		+++
Flavonoids		++
Tannins	Catechic tannins	++
	Gallic tannins	++
Anthracene derivatives	Quinones	+
	O-Heterosides	+
	C-Heterosides	+
Saponosides		+++
Oses et holosides		-
Alkaloids	Dragendorff	-
	Mayer	-

Category: Strong presence: +++; average presence: ++; low presence: + and absent: -.

### 3.3 Extraction and quantitative analysis of phenolic compounds

#### 3.3.1 Extraction yields

**FIGURE 2:**
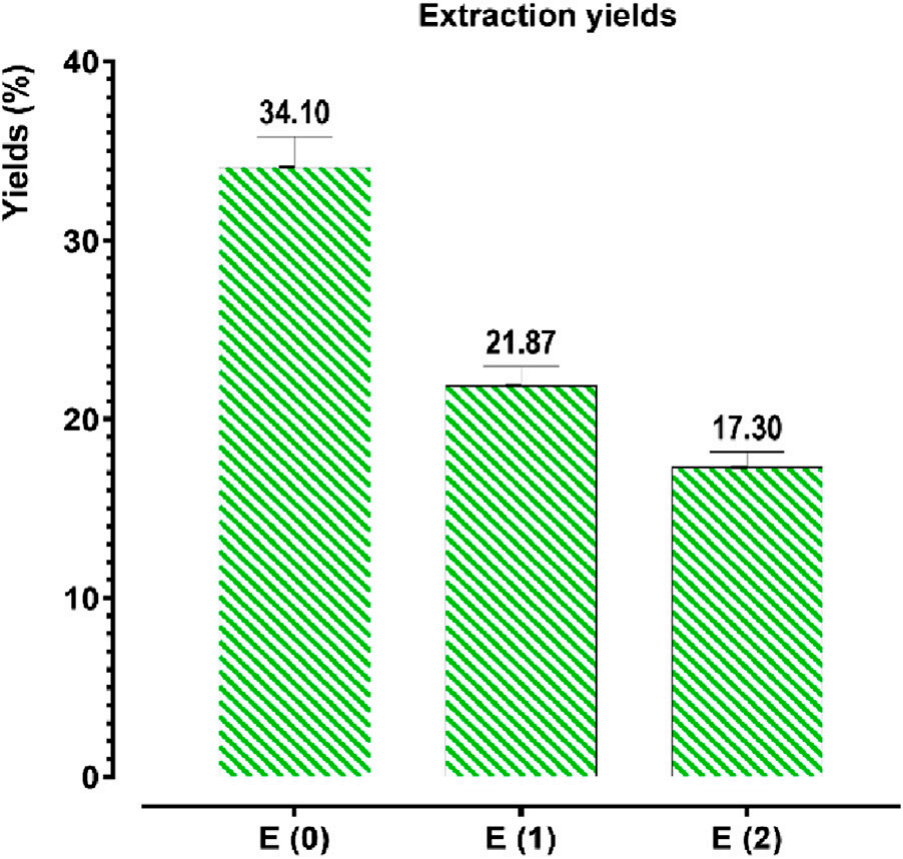
Extraction yields of phenolic compounds from *Juglans regia*. E(0): Decoction; E(1): Aqueous extract obtained by Soxhlet; E(2): Hydroethanolic extract obtained by Soxhlet. The values represent the extraction yields (%) of phenolic compounds for each extraction method.

The extraction of phenolic compounds from *J. regia* husks was carried out using three distinct methods: decoction (E0), Soxhlet extraction with water (E1), and Soxhlet extraction with ethanol-water (E2). The results presented in Figure 2 reveal that decoction (E0) achieved the highest extraction efficiency, yielding 34.10%, significantly higher than the Soxhlet extraction methods, which yielded 21.87% for E1 and 17.30% for E2. These findings highlight that decoction, owing to its milder and more prolonged extraction process, enhances the release of phenolic compounds, making it a considerably more efficient technique than Soxhlet-based methods. The superior performance of decoction may be attributed to gradual cell wall disruption, allowing for optimal solubilization of phenolics. In contrast, Soxhlet extraction, despite its continuous solvent reflux, may lead to thermal degradation or reduced solubility of certain phenolic constituents.

#### 3.3.2 Determination of concentrations of polyphenols, flavonoids, condensed tan-nins, and hydrolysable tannins

**FIGURE 3:**
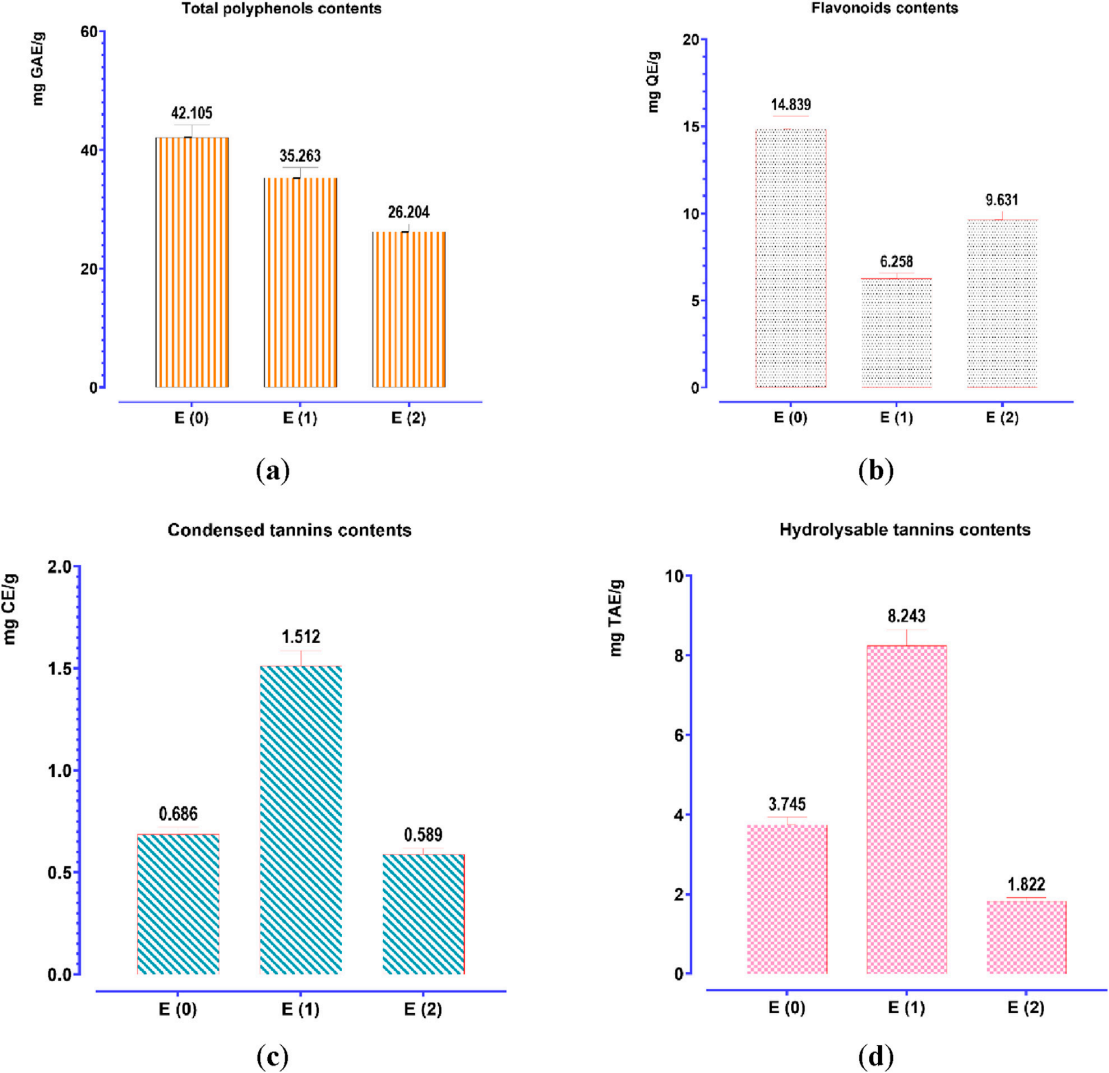
Contents of phenolic compounds: **(a)** total polyphenols; **(b)** flavonoids; **(c)** condensed tannins; **(d)** hydrolyzable tannins. Mean values ± standard deviations of determinations performed in triplicate are reported. Means are significantly different (*p < 0.001*).

To quantify the polyphenol, flavonoid, condensed tannin, and hydrolyzable tannin concentrations in the aqueous (E0) and hydroethanolic (E1, E2) extracts of *J. regia* husks, calibration curves were established using gallic acid (Y = 0.095X + 0.003; *R* ^2^ = 0.998), quercetin (Y = 0.073X − 0.081; *R* ^2^ = 0.995), catechin (Y = 0.742X + 0.032; *R* ^2^ = 0.998), and tannic acid (Y = 0.1700X − 0.0007; *R* ^2^ = 0.996). The total content of polyphenols, flavonoids, condensed tannins, and hydrolyzable tannins was expressed in milligrams equivalent of gallic acid (mg GAE/g), quercetin (mg QE/g), catechin (mg CE/g), and tannic acid (mg TAE/g) per Gram of extract, respectively. The analytical results (Figure 3) indicate a significant variation in the concentration of these bioactive compounds depending on the extraction method. Decoction (E0) yielded the highest polyphenol (42.105 mg GAE/g) and flavonoid (14.888 mg QE/g) contents, significantly surpassing those obtained via Soxhlet extraction, whether using water (E1) or a water-ethanol mixture (E2). This suggests that decoction optimally preserves and extracts polyphenols and flavonoids, likely due to its prolonged extraction time at controlled temperatures, enhancing compound solubility and bioavailability. Conversely, the Soxhlet extraction method using water (E1) yielded the highest concentrations of condensed tannins (1.512 mg CE/g) and hydrolyzable tannins (8.243 mg TAE/g), indicating its superior efficiency in extracting tannin-rich fractions. In contrast, the hydroethanolic extract (E2) exhibited the lowest concentrations for all analyzed compounds, suggesting that ethanol-water mixtures may not be as effective in extracting these specific metabolites from *J. regia* husks. These findings confirm that the extraction method significantly influences the chemical composition of the resulting extracts, with decoction favoring the recovery of polyphenols and flavonoids. In contrast, Soxhlet extraction with water enhances tannin extraction. Understanding these methodological differences is crucial for optimizing bioactive compound recovery based on the targeted pharmacological or nutraceutical application.

#### 3.3.3 Analysis and identification of polyphenols in *Juglans regia* extract by high-pressure liquid chromatography–mass spectrometry (HPLC/MS)

**FIGURE 4:**
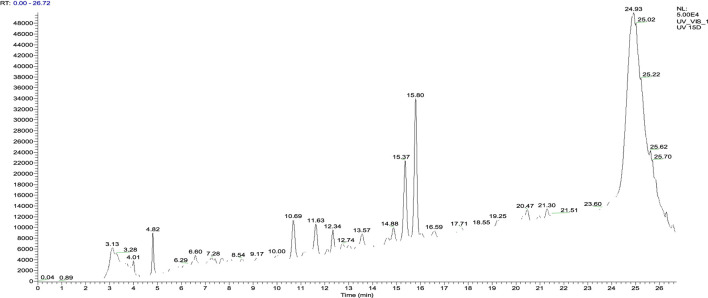
HPLC chromatograms of compounds extracted from the decocted *Juglans regia*.

*Juglans regia* decoctions were analyzed using HPLC/MS. The chromatogram presented in Figure 4 highlights the compounds detected in the husks of *J. regia*. Through a detailed examination of the mass spectra in conjunction with the chromatogram, 23 molecules were identified, summarized in Table 8.

**TABLE 8:** List of Compounds Identified by Mass Spectrometry in the husks of *Juglans regia*.

RT (min)	Molecules	Classes	Exact masses	[M-H]^−^ (m/z)	Fragment ions (m/z)	Area %
3.13	Gallic acid	Phenolic acid	170	169	169–125	5.18
4.01	Chlorogenic acid	Phenolic acid	354	353	353- 191-179	1.04
4.82	Ferulic acid	Phenolic acid	194	193	178-149-134	2.7
6.29	Isorhamnetin	Flavonoid	316	315	315-285-147	0.31
6.60	Quinic acid	Phenolic acid	192	191	191 - 127	1.09
7.28	(−) Epicatechin	Flavonoid	290	289	245 - 205 - 179	0.22
7.70	Ferulic acid hexoside	Phenolic acid	356	355	175 - 193 - 161	0.57
8.54	Epigallocatechin	Flavonoid	306	305	179 - 221 - 125	0.3
10.69	Gallotannin	Polyphenol	666	665	665-407-335-113	4.59
11.14	Protocatechuic acid	Phenolic acid	154	153	153-109-93	0.5
11.63	Hydrojuglone derivative rhamnoside	Phenolic compounds	450	449	449 - 303 - 285	3.5
12.34	Syringaldehyde	Phenolic compounds	182	181	181-166-151-123	2.33
13.57	Juglone	Phenolic compounds	174	173	173-145-117	1.87
14.63	Quercitrin	Flavonoid	448	447	301-255-243-179	1.07
14.88	p-Coumaric acid derivative	Phenolic acid	282	281	163 - 135 - 119	2.03
15.37	Regiolone	Phenolic compounds	178	177	175-159-131	9.25
15.80	Hydrojuglone glucoside	Phenolic compounds	338	337	337-175	14.51
19.25	Juglanin B	Flavonoid	328	327	312 - 253	0.46
20.47	Ellagic Acid	Phenolic acid	302	301	283 - 257 - 229 - 185	1.22
20.96	Casuarictin	Polyphenol	936	935	935-633-301	0.36
21.30	Hydrojuglone	Phenolic compounds	176	175	175-147-115	0.93
24.93	Pedunculagin	Polyphenol	784	783	783-301-275-257-229	45.12
26.29	Ferulic acid derivative	Phenolic acid	522	521	473 - 503 - 337	0.82

The analysis conducted using HPLC/MS in negative mode enabled the identification and structural characterization of 23 compounds, including phenolic compounds, flavonoids, and polyphenols, present in the decoction of *J. regia*. This identification was primarily based on the ions [M−H]^-^ and their characteristic fragments. Polyphenols were the most abundant class, followed by phenolic compounds, phenolic acids, and flavonoids, as outlined in Table 9. These findings highlight the exceptional richness of *J. regia* in bioactive secondary metabolites, such as hydrolyzable tannins like pedunculagin and phenolic acids like gallic acid. These compounds, widely recognized for their antioxidant and antimicrobial properties, underscore the plant’s significant potential for applications in nutrition, health, and cosmetics.

**TABLE 9:** The percentages of the phenolic compounds classes identified in the *Juglans regia* decoction.

Classes	Percentages (%)
Flavonoids	2.36
Phenolic acids	15.15
Phenolic compounds	32.39
Polyphenols	50.07
Total	99.97

Among the significant compounds, pedunculagin, a hydrolyzable tannin accounting for 45.12% of the total content, is notable for its exact mass of 784 Da. It is characterized by an ion [M−H]^-^ at *m/z* 783 and fragment ions at *m/z* 301, 275, 257, and 229, resulting from the loss of galloyl groups and water or carbon dioxide molecules. This fragmentation mechanism reflects the structural complexity of this compound. Similarly, hydrojuglone glucoside, representing 14.51%, exhibits an ion [M−H]^-^ at *m/z* 337 and a fragment at *m/z* 175, attributed to the cleavage of the glycosidic bond. These features highlight the chemical diversity of the metabolites present.

Additionally, other notable compounds were identified. For instance, regiolone, a naphthoquinone representing 9.25%, has an exact mass of 178 Da and an ion [M−H]^-^ at *m/z* 177, accompanied by fragments at *m/z* 175, 159, and 131, resulting from successive losses of methyl and hydroxyl groups. Gallotannin, constituting 4.59% of the total content, has an exact mass of 666 Da. Its ion [M−H]^-^ at *m/z* 665 generates fragments at *m/z* 407, 335, and 113, reflecting the loss of galloyl groups and the degradation of phenolic residues. Furthermore, the rhamnoside derivative of hydrojuglone (3.5%) is characterized by an exact mass of 450 Da and an ion [M−H]^-^ at *m/z* 449, with fragments at *m/z* 303 and 285, resulting from the loss of the rhamnoside group and the dehydration of hydrojuglone, respectively.

Though present in smaller proportions, several other compounds also merit mention due to their bioactive properties. For example, gallic acid (5.18%) shows an ion [M−H]^-^ at *m/z* 169 and a fragment at *m/z* 125, attributed to a decarboxylation mechanism. Similarly, chlorogenic acid (1.04%) exhibits an ion [M−H]^-^ at *m/z* 353 and fragments at *m/z* 191 and 179, resulting from the cleavage of the ester bond between quinic acid and caffeic acid. Ferulic acid (2.7%) is distinguished by an ion [M−H]^-^ at *m/z* 193 and fragments at *m/z* 178, 149, and 134, reflecting successive losses of methoxy and hydroxyl groups.

This in-depth analysis of mass spectra reveals specific fragmentation mechanisms for each class of compounds, emphasizing the richness and complexity of the secondary metabolites found in *J. regia* extracts. These findings, which highlight the plant’s bioactive potential, pave the way for diverse and promising applications in health and nutrition.

**FIGURE 5:**
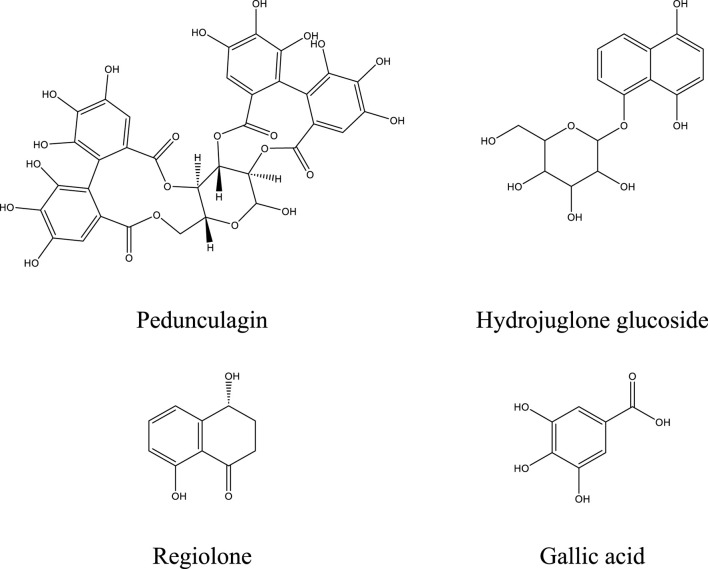
Structure of the major compounds.

Figure 5 illustrates the chemical structures of the main identified compounds and enriches this analysis by providing precise details about their configuration.

### 3.4 Antioxidant activity

**FIGURE 6:**
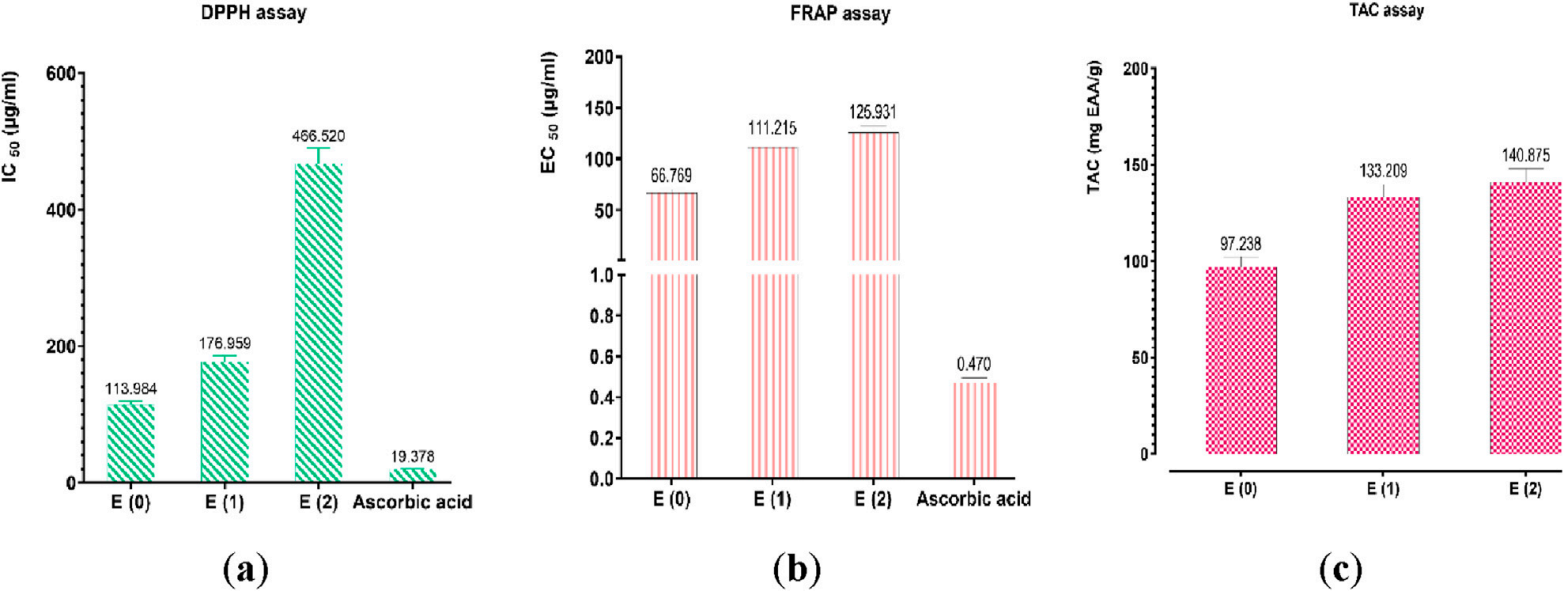
Antioxidant activity of ascorbic acid and extracts by **(a)** DPPH, **(b)** FRAP, and **(c)** TAC assays. Mean values ± standard deviations of determinations performed in triplicate are reported. Means are significantly different (*p < 0.001*).

**FIGURE 7:**
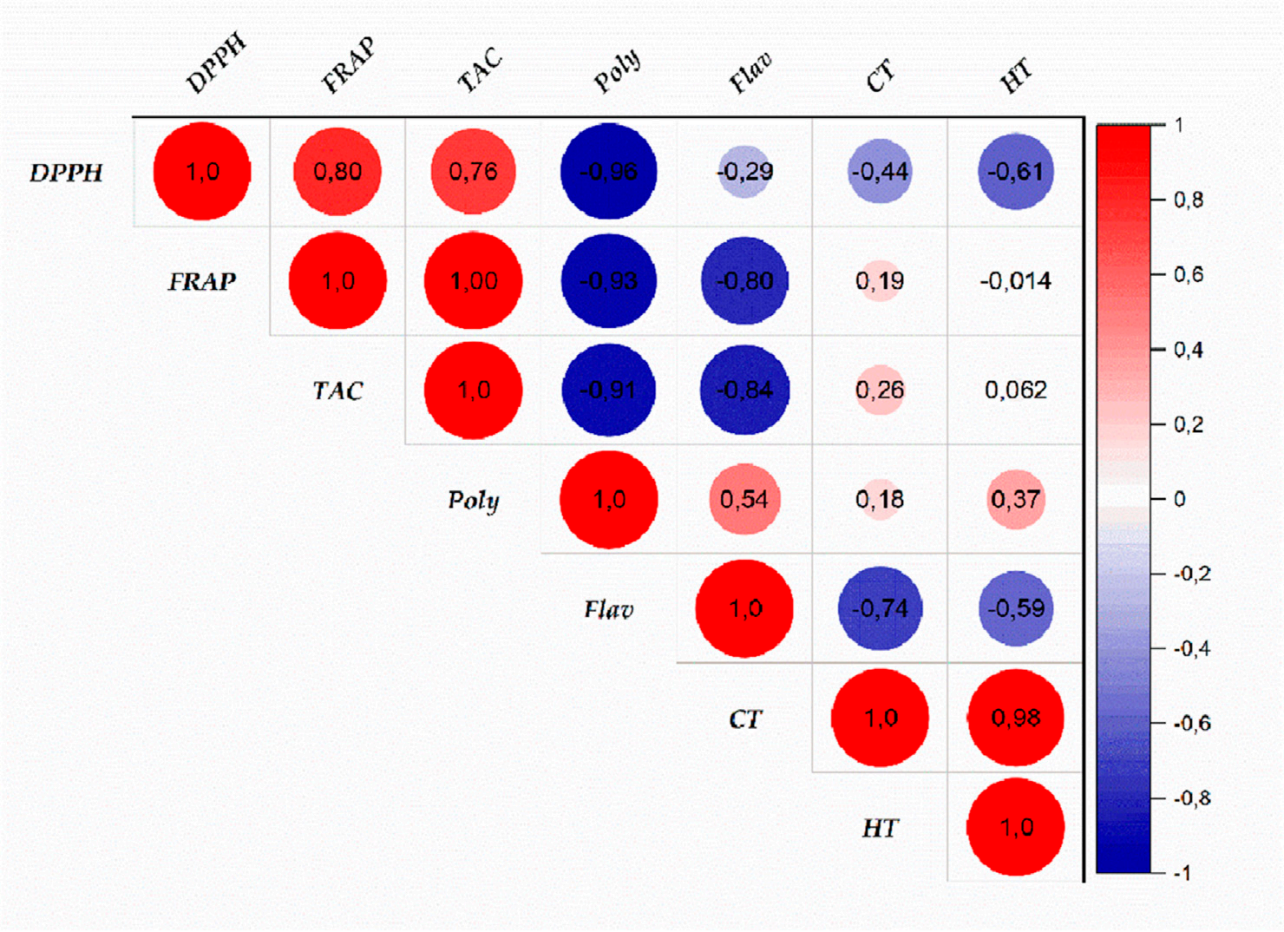
Correlation between antioxidant activities and phenolic compound contents of *Juglans regia* extracts. (Poly: polyphenols; Flav: flavonoids; CT: condensed tannins; HT: hydrolyzable tannins).

The antioxidant potential of the hydroethanolic E(2) and aqueous (E0, E1) extracts of *J. regia* husks was assessed using three complementary techniques: DPPH (radical scavenging activity), FRAP (ferric reducing antioxidant power), and TAC (total antioxidant capacity). Ascorbic acid was employed as a reference antioxidant, and calibration curve formulas derived for each method were as follows: FRAP (Y = 0.0048X + 0.0974; *R* ^2^ = 0.8963), TAC (Y = 0.0407X + 0.0211; *R* ^2^ = 0.9949), and DPPH (Y = 1.0130X − 8.0320; *R* ^2^ = 0.9893). The results confirmed the high antioxidant potential of the tested extracts by demonstrating their substantial capacity to scavenge free radicals and reduce oxidative processes (Figure 6). Among the tested extracts, E0 exhibited the most potent antioxidant activity, with EC_50_ values of 113.984 μg/mL (DPPH) and 66.769 μg/mL (FRAP), suggesting a high concentration of bioactive compounds contributing to its superior efficacy. Meanwhile, the TAC assay revealed that extract E(2) had the highest total antioxidant capacity (140.875 mgEAA/g). These results indicate that different extraction methods influence the antioxidant properties of the extracts, likely due to variations in phenolic compound content and composition. The extracts displayed promising antioxidant activity, though at higher effective concentrations compared to ascorbic acid (IC_50_ = 19.378 μg/mL for DPPH and EC_50_ = 0.470 μg/mL for FRAP). The discrepancies among the DPPH, FRAP, and TAC methods emphasize the necessity of employing multiple methodologies to comprehensively evaluate antioxidant activity. A strong correlation was observed between the levels of phenolic compounds (polyphenols, flavonoids, condensed and hydrolyzable tannins) and antioxidant capacity, reinforcing the crucial role of these bioactive constituents in free radical neutralization. Figure 7 highlights intricate relationships between phenolic content and antioxidant assays, with the FRAP and TAC methods showing a strong positive correlation (r = 1.00). In contrast, polyphenols exhibited significant negative correlations with these tests, suggesting specific interactions or methodological limitations. Interestingly, condensed and hydrolyzable tannins were highly correlated (r = 0.98), implying a similar contribution to antioxidant effects. These findings underscore the importance of phenolic compounds in antioxidant mechanisms, with varying influences depending on the analytical method used. Ultimately, the results highlight the value of a multi-method approach in obtaining a comprehensive and reliable assessment of antioxidant properties in plant extracts.

### 3.5 Antimicrobial activity

The decoction extract of *J. regia* exhibits intriguing antibacterial action against a range of pathogens, including yeasts, molds, and Gram-positive and Gram-negative bacteria (GPC and GNB), according to the analysis of the results shown in Table 10. Although this extract’s efficiency is typically lower than that of reference antibiotics and antifungals, it shows promise in certain situations when tested by minimum inhibitory concentrations (MIC) and bactericidal or fungicidal concentrations (MBC/MFC). With MICs ranging from 300 μg/mL for *E. faecalis* to 5000 μg/mL for *Staphyloccocus epidermidis* and methicillin-resistant *Staphylococcus aureus* (MRSA), *J. regia* therefore exhibits noteworthy activity for Gram-positive bacteria. Sensitive bacteria, such as *E. faecalis*, show notable effectiveness with comparatively low MIC and MBC (300 and 600 μg/mL, respectively). The extract exhibits more targeted efficacy against Gram-negative bacteria, namely, against *Shigella* sp. (MIC = 150 μg/mL) and *A. baumannii* (MIC = 150 μg/mL), which are pathogens of interest because of their growing resistance to traditional antibiotics. Conversely, the MIC for organisms like *Klebsiella pneumoniae* and *E. coli* surpasses 5000 μg/mL, indicating reduced efficacy in these situations. Furthermore, the MIC of 2,500 μg/mL for *C. albicans* and 2,500–5000 μg/mL for other *Candida* species demonstrates the extract of *J. regia*’s intriguing antifungal activity. With MIC and MFC of 2,500 and 5000 μg/mL, respectively, molds like *A. niger* also show moderate sensitivity. Antibiotics and the reference antifungal, terbinafine, on the other hand, typically exhibit much lower MICs, indicating their higher efficacy. However, for pathogens that are resistant to traditional therapies, the extract of *J. regia* may offer a natural substitute or a therapeutic addition. The significance of additional investigation into this extract’s bioactive components, modes of action, and potential for the creation of novel antimicrobial drugs is underscored by these findings.

**TABLE 10:** The MIC, MBC, and MFC (µg/mL) values of the *Juglans regia* decoction extract, along with the MIC values of antibiotics and antifungal agents.

Microorganism		*J. regia*		Antibiotics*					Antifungals ^#^
		MIC	MBC or MFC	Gentamycin	Amoxicillin–Clavulanate	Vancomycin	Trimethoprim–Sulfamethoxazole	Penicillin G	Terbinafine
*GPC*	*S. epidermidis*	5000	5000	2		>8	>4/76		
	*S. aureus BLACT*	2,500	2,500	<0.5		2	<10		
	*S. aureus STAIML/MRS/mecA/HLMUP/BLACT*	5000	5000	2		>8	>4/76		
	*S. acidominimus*	1200	2,500	≤250		<0.5		0.03	
	*S. group D*	5000	5000	>1000		<0.5		0.13	
	*S. agalactiae (B)*	1200	2,500	≤250		>4		0.06	
	*S. porcinus*	2,500	2,500	≤250		<0.5		0.06	
	*E. faecalis*	300	600	≤500		1	≤0.5/9.5		
	*E. faecium*	5000	5000	≤500		>4	>4/76		
*GNB*	*A. baumannii*	150	300	≤1	≤2/2		≤1/19		
	*A. baumannii 2,410*	300	300						
	*E. coli*	>5000	>5000	2	8/2		≤1/19		
	*E. coli ESBL*	>5000	>5000	2	>8/2		>4/76		
	*E. coli ESBL 5765*	>5000	>5000						
	*E. aerogenes*	2,500	5000	≤1	8/2		≤1/19		
	*E. cloacae*	2,500	2,500	>4	>8/2		>4/76		
	*E. cloacae 2,280*	2,500	5000						
	*C. koseri*	2,500	5000	<1	>8/2		<20		
	*K. pneumoniae*	5000	5000	≤1	≤2/2		≤1/19		
	*K. pneumoniae 1015*	5000	>5000						
	*P. mirabilis*	1200	2,500	2	≤2/2		>1/19		
	*P. aeruginosa*	5000	5000	2	>8/2		4/76		
	*P. aeruginosa 1124*	5000	>5000						
	*P. fluorescence*	2,500	5000	4	>8/2		4/76		
	*P. putida*	2,500	2,500	>4	>8/2		>4/76		
	*S. marcescences*	2,500	5000	4	>8/2		>4/76		
	*Sallemonella sp*	600	1200	>4	8/2		>4/76		
	*Shigella sp*	150	300	>4	8/2		>4/76		
	*Y. enterolitica*	600	1200	≤1	8/2		2/38		
*Yeasts*	*C. albicans*	2,500	5000						12.500
	*C. kefyr*	5000	5000						25.000
	*C. krusei*	2,500	2,500						50.000
	*C. parapsilosis*	2,500	2,500						6.250
	*C. tropicalis*	>5000	>5000						12.500
	*C. dubliniensis*	5000	5000						3.125
	*S. cerevisiae*	5000	5000						3.125
*Molds*	*A. niger*	2,500	5000						3.125

*The antibiotics’ MIC (µg/mL) was determined by the BD, Phoenix™ identification and antibiogram instrument; #: The MIC (µg/mL) of terbinafine was determined on a microplate.

### 3.6 Anticoagulant activity

**FIGURE 8:**
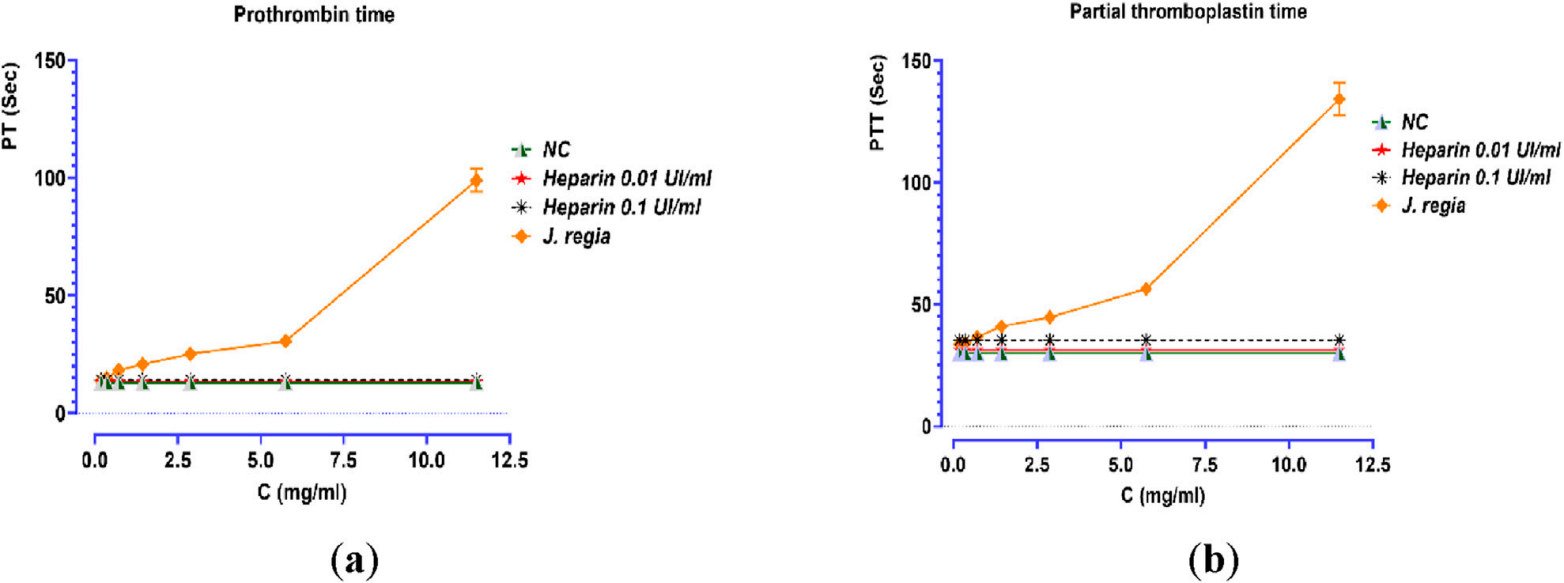
Effect of *Juglans regia* decocted extract (E_0_), standard control (NC), and heparin on prothrombin time **(a)** and partial thromboplastin time **(b)**.

The prothrombin time (PT) and activated partial thromboplastin time (aPTT) assays, two key indicators of coagulation activity, revealed a strong dose-dependent effect (*p < 0.001*) following treatment with the *J. regia* decoction (Figure 8). The extract induced a progressive and significant prolongation of coagulation times, particularly at higher concentrations, when compared to the negative control (NC) and samples treated with low doses of heparin (0.01 UI/mL and 0.1 UI/mL). At a concentration of 11.5 mg/mL, PT values increased from 13 s (NC) to 98.9 s, marking a substantial elevation, in contrast to the minimal variations observed with heparin (ranging from 13.4 to 14.1 s). Similarly, aPTT values showed a significant increase, rising from 30 s (NC) to 134.2 s at the highest extract concentration. Although the precise mechanism of action remains to be fully elucidated, these findings strongly suggest that *J. regia* exerts a notable anticoagulant effect, likely through interactions with coagulation factors. Interestingly, the extract exhibited a more pronounced dose-dependent response compared to heparin, which demonstrated consistent but moderate anticoagulant efficacy in this study. These results highlight the potential of *J. regia* as a natural anticoagulant agent, warranting further research to explore its safety profile, therapeutic potential, and underlying molecular mechanisms.

### 3.7 Antidiabetic activity

#### 3.7.1 Evaluation of the inhibitory effect of decocted extract on the activity of α-amylase and α-glucosidase, *in vitro*

**FIGURE 9:**
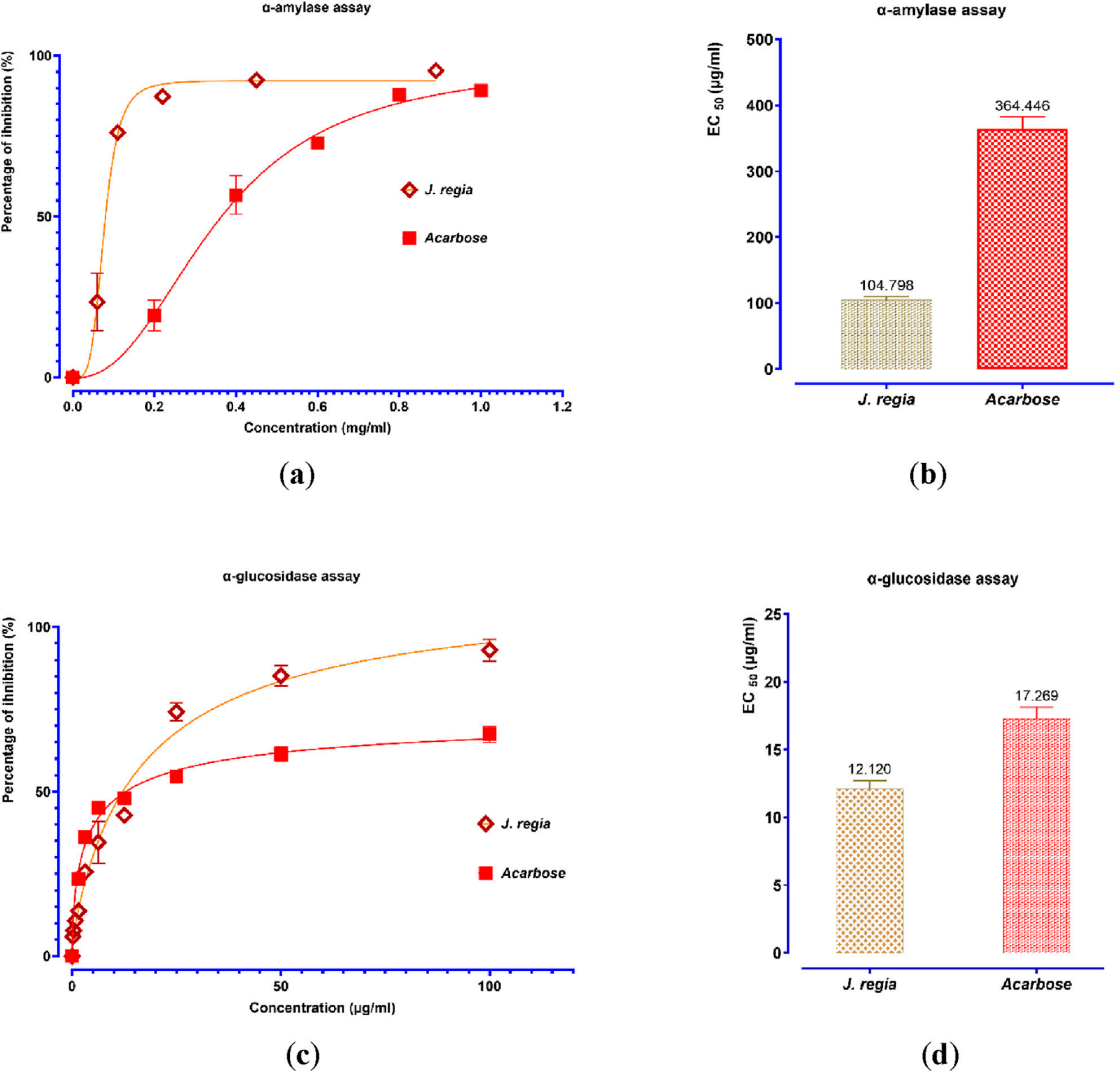
Percent inhibition and EC_50_ of inhibitory effects on α-amylase **(a,b)** and α-glucosidase **(c,d)** activities by *Juglans regia* decocted extract and acarbose, *in vitro*. Values are means ± SEM (n = 3).

The decocted extract of *J. regia* exhibits significant antidiabetic potential, as demonstrated by its strong inhibitory effects on the enzymatic activities of α-amylase and α-glucosidase *in vitro* (Figure 9). A dose-dependent inhibition of α-amylase activity was observed, with a maximal inhibition rate of 94.39% at 0.89 mg/mL, closely resembling that of acarbose (96.23%), a well-established α-amylase inhibitor. Notably, *J. regia* demonstrated greater efficacy at lower concentrations, as indicated by its significantly lower EC_50_ value (104.798 μg/mL) compared to acarbose (364.446 μg/mL), suggesting a higher potency per unit concentration. Similarly, the *J. regia* extract exhibited a dose-dependent inhibition of α-glucosidase activity, reaching 90.57% inhibition at 100 mg/mL, slightly lower than acarbose (95.34%). However, its EC_50_ value (12.12 μg/mL) was notably lower than that of acarbose (17.27 μg/mL), indicating greater inhibitory potency at lower doses. These findings strongly suggest that the decocted extract of *J. regia* interacts selectively and effectively with carbohydrate-hydrolyzing enzymes, making it a promising natural alternative to synthetic enzyme inhibitors for the management of type 2 diabetes. Further investigations are warranted to identify the bioactive compounds responsible for these effects and to elucidate their mechanisms of action, particularly in relation to enzyme binding affinity, inhibition kinetics, and metabolic modulation *in vivo*. These findings provide a scientific basis for the therapeutic potential of *J. regia* in glycemic control, emphasizing the need for additional pharmacological and clinical studies to validate its efficacy and safety.

#### 3.7.2 Evaluation of the inhibitory effect of decocted extract on the activity of α-amylase and α-glucosidase, *in vitro*

The acute toxicity assessment of the decocted extract of *J. regia* revealed that it is non-toxic, even at a high dose of 2 g/kg. Throughout the study period, no adverse effects, including diarrhea, vomiting, abnormal mobility, or mortality, were observed following oral administration of the extract. These findings suggest a high safety margin, supporting the potential therapeutic use of *J. regia* without immediate toxicological risks.

#### 3.7.3 Study of the antihyperglycemic activity of *Juglans regia* decocted extract in normal rats *in vivo*

**FIGURE 10:**
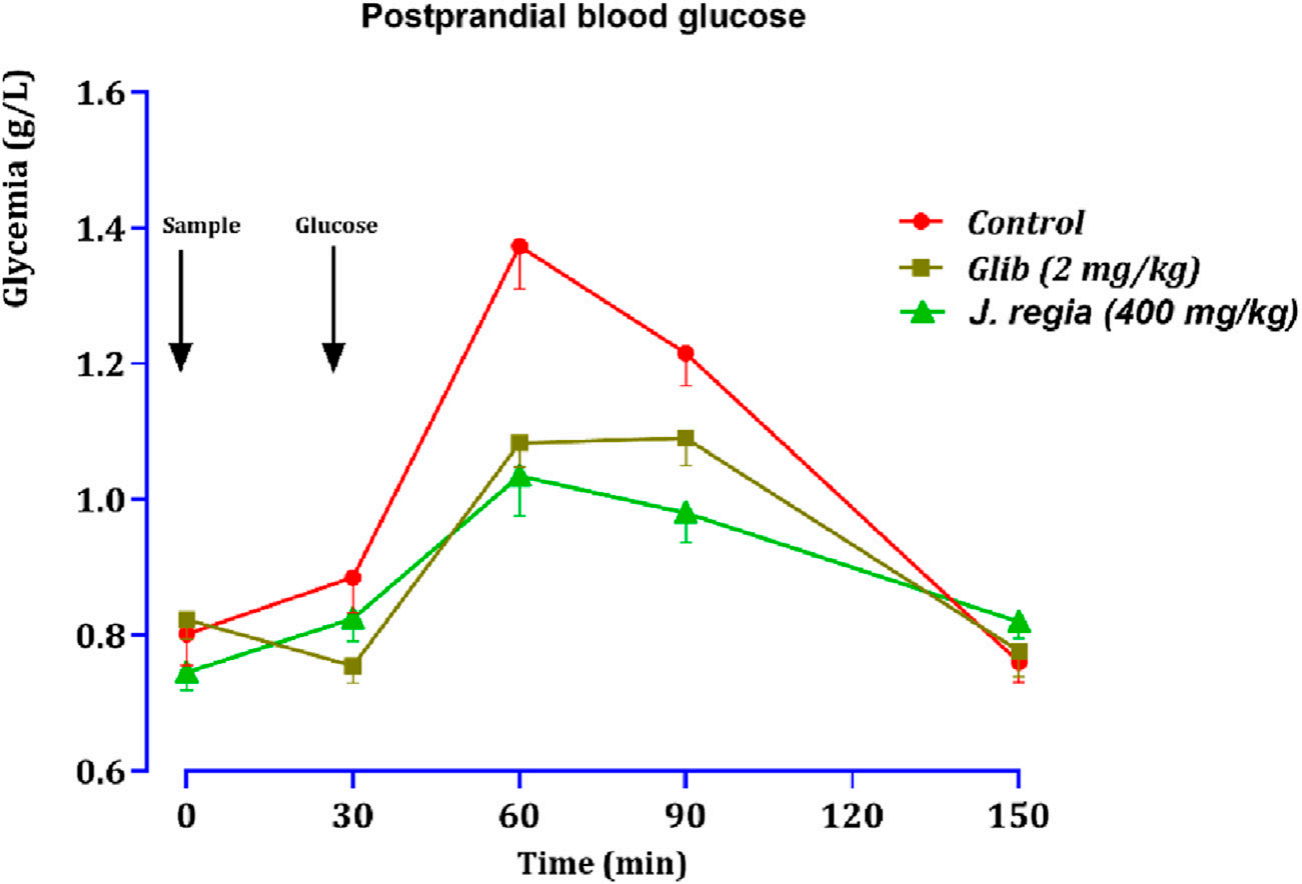
Variation in postprandial blood glucose in normal rats after administration of test products (decocted extract and glibenclamide). Values are means ± SEM. (n = 6). *p < 0.01* in comparison with the control.

**FIGURE 11:**
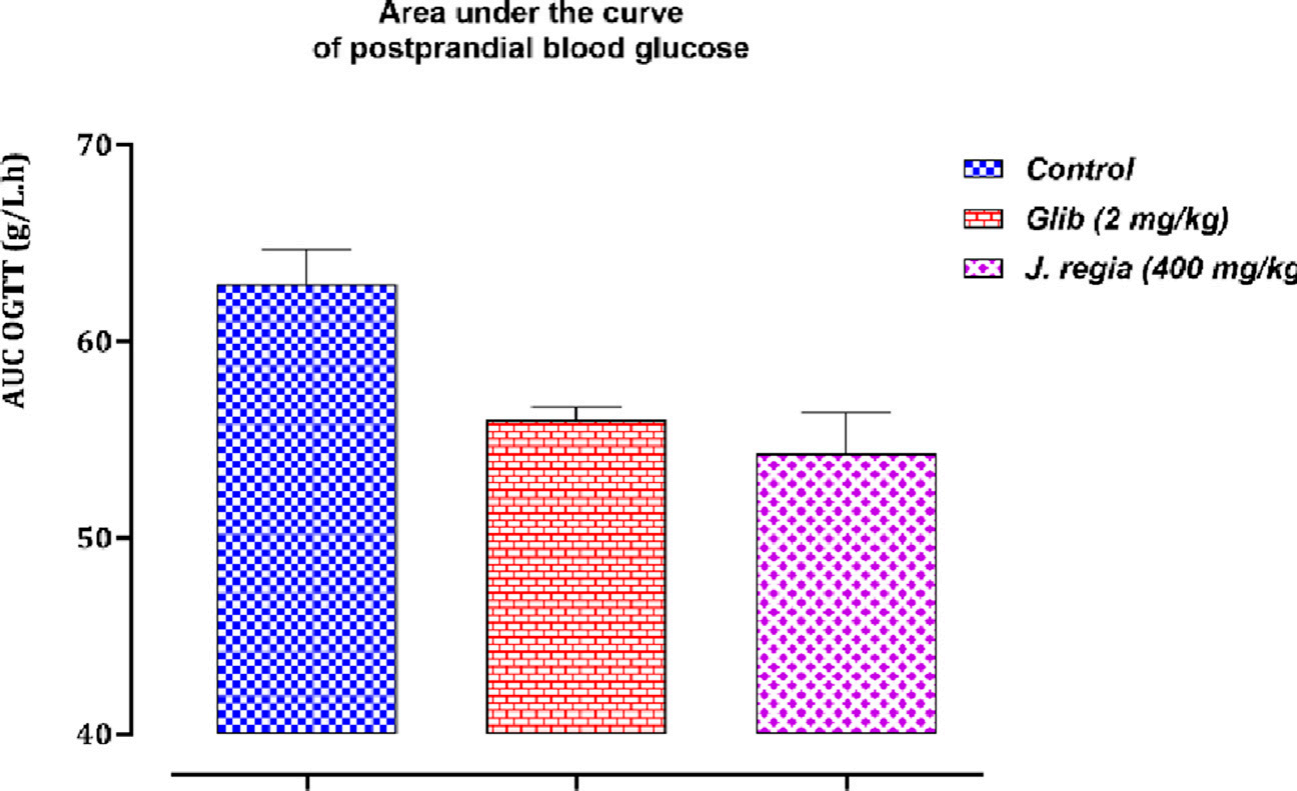
Variation in the area under the curve of postprandial blood glucose in normal rats after administration of tested products (decocted extract and glibenclamide). Values are means ± SEM. (n = 6). p < 0.05 when compared with control.

Figures 10, 11 compare the total areas under the blood glucose curve during the 150 min and analyze the glucose tolerance test.

##### 3.7.3.1 Test of oral glucose tolerance

Thirty minutes after glucose loading, normal rats exhibited a sharp peak in blood glucose levels. However, rats treated with glibenclamide or the decocted *J. regia* extract showed a favorable response to glucose loading. Compared to the control group that received distilled water, oral administration of 400 mg/kg of *J. regia* extract significantly reduced postprandial hyperglycemia 30 min before glucose overload (Figure 10). Glibenclamide also significantly decreased postprandial hyperglycemia within the first hour (60 min) after glucose overload (*p < 0.001*; 1.08 g/L), in contrast to the distilled water-treated group.

As illustrated by the postprandial glucose graph, data analysis highlights significant differences between the control group, the glibenclamide-treated group (2 mg/kg), and the *J. regia*-treated group (400 mg/kg). The control group experienced a rapid rise in blood sugar, peaking at 1.45 g/L at 60 min and gradually declining. In contrast, glibenclamide treatment markedly reduced this increase, resulting in a lower glycemic level of approximately 1.16 g/L simultaneously. Remarkably, the *J. regia* extract demonstrated comparable, if not superior, efficacy, reducing postprandial glucose elevation to around 1.0 g/L at 60 min, followed by a rapid and pronounced decline.

These findings underscore the potent hypoglycemic effect of the *J. regia* extract, which effectively lowers postprandial blood glucose levels and rivals the well-established antidiabetic agent glibenclamide. Furthermore, the rapid decline in blood glucose levels observed in the *J. regia* treated group suggests enhanced metabolic regulation. These results support the potential of *J. regia* as a natural alternative for managing postprandial hyperglycemia. Nonetheless, further studies are needed to validate these *in vivo* findings and elucidate the molecular mechanisms underlying this effect.

##### 3.7.3.2 Areas under the curve (AUCs) of postprandial glucose levels

Analysis of the data related to the area under the curve (AUC) for postprandial blood glucose (Figure 11) reveals significant differences among the three groups under study: the control group, the glibenclamide-treated group (2 mg/kg), and the *J. regia* -treated group (400 mg/kg). The average AUC values indicate that postprandial blood glucose levels in the treated groups were significantly lower than in the control group. Specifically, the glibenclamide-treated group exhibited average AUC values ranging from 53.36 to 57.72 g/L.h, while the *J. regia*-treated group showed values between 47.72 and 60.32 g/L.h. In contrast, the control group displayed higher average AUC values, ranging from 59.72 to 71.28 g/L.h. These results highlight the hypoglycemic efficacy of both treatments, with the *J. regia*-treated group showing a more pronounced reduction in several parameters, suggesting an effect comparable to or surpassing that of glibenclamide. This indicates that the *J. regia* extract significantly impacts carbohydrate metabolism, effectively mitigating postprandial glycemic spikes. These findings further support the potential of *J. regia* as a phytotherapeutic option for managing hyperglycemia, particularly in type 2 diabetes. However, additional research is required to explore these observations in greater detail and to uncover the molecular mechanisms underlying this activity.

### 3.8 Molecular docking

The main components in *J. regia*’s decoction showed encouraging interactions with several biological targets with antibacterial, antifungal, antioxidant, antidiabetic, and antithrombotic properties, according to molecular docking research (Table 11). Pedunculagin and Hydrojuglone glucoside stand out among the examined ligands with exceptionally high binding affinities, indicating a powerful therapeutic potential. Pedunculagin, for instance, has a strong affinity for thrombin heavy chain (−8.9 kcal/mol) and Acriflavine resistance protein B (−10.1 kcal/mol), whilst Hydrojuglone glucoside has strong affinities for D-alanine-D-alanine ligase (−9.6 kcal/mol) and NADPH oxidase (−9.2 kcal/mol). These findings suggest that these substances may function as strong antibacterial agents by blocking essential proteins implicated in the manufacture of bacterial cell walls and antibiotic resistance, as well as antioxidant agents by lowering the generation of free radicals. Comparable affinities for vital enzymes like CYP51 VARIANT1 (−8.0 kcal/mol) and Lanosterol 14-alpha demethylase (−8.0 and −7.6 kcal/mol, respectively) are demonstrated by Pedunculagin and Hydrojuglone glucoside in terms of their antifungal activities, indicating a possible inhibition of ergosterol biosynthesis, a crucial component of the fungal membrane. Alpha-amylase (−8.0 and −8.3 kcal/mol) and alpha-glucosidase (−8.0 and −7.5 kcal/mol) are both efficiently inhibited by these two chemicals for antidiabetic effects, which may help control blood sugar levels by modifying the digestion of carbohydrates. Lastly, by preventing blood coagulation, pedunculagin’s high affinity for thrombin (−8.9 kcal/mol) raises the possibility of an antithrombotic effect. Despite often having lower binding affinities, the other chemicals, gallic acid and regiolone, continue to be of moderate relevance for some targets. Gallic acid exhibits a mild affinity for specific antioxidant targets such as 1OG5 (−5.4 kcal/mol). Still, regiolone exhibits a substantial affinity for the protein 2ZDQ (−8.1 kcal/mol) in the context of antibacterial activity. These findings imply that these substances might complement one another in formulations that work in concert.

**TABLE 11:** Protein targets and molecular docking parameters.

Activities	Targets \ ligands	Pedunculagin	Hydrojuglone glucoside	Regiolone	Gallic acid
Antibacterial activity	1JZQ	−9.3	−7.7	−6.2	−5.4
	1KZN	−8.5	−7.5	−6	−5.8
	2VEG	−7.2	−6.7	−5.1	−5.3
	2W9G	−6.4	−8.2	−6.7	−5.5
	2ZDQ	−2.2	−10	−8.1	−7
	3SRW	−6	−9	−6.7	−5.7
	3UDI	−6.3	−8	−6	−5.5
	4URN	−8.7	−7.1	−6	−6
	4ZBE	−8.4	−7.5	−6.7	−6.3
	5J8G	−6.2	−5.8	−4.7	−4.7
	5OE3	−8	−7.9	−6.6	−6.7
	2CAG	−6.6	−9.6	−7.7	−7.1
	4DX5	−10.1	−8	−7.2	−5.9
	4HL2	−8.5	−7.9	−6.6	−6.1
	3VOB	−8.5	−8.4	−7.7	−6.6
Antifungal activity	5FSA	−8	−8	−7.3	−6
	5V5Z	−8	−7.6	−6.6	−5.9
Antioxidant activity	1N8Q			−4	−5.2
	1OG5	−5.6	−8.3	−6.6	−5.4
	2CDU	−8.2	−9.2	−7	−6.7
	3NRZ	-	−5.4	−5.4	−7.3
	1HL5	−7.5	−7.3	−6.4	−5.7
	2F8A	−5.6	−5.9	−4.6	−4.1
Antidiabetic activity	4W93	−8	−8.3	−5.9	−5.4
	3W37	−8	−7.5	−6.1	−6.4
Anticoagulant activity	4UFD	−8.9	−8	−6.5	−6.5

These results suggest that the compounds in the decoction of *J. regia*, particularly Pedunculagin and Hydrojuglone glucoside, could be exploited for their antimicrobial, antioxidant, and metabolic properties. The in-depth analysis of the molecular interactions of pedunculagin with its biological targets reveals precise reaction mechanisms and highlights the amino acid residues involved in these interactions (Table 12). For the target 2CDU (NADPH Oxidase), pedunculagin binds to the active pocket containing FAD, forming hydrogen bonds with key residues such as Arg91 and Tyr198 while establishing hydrophobic interactions with Phe115 and Leu294. This binding inhibits the production of superoxide radicals, thereby reducing oxidative stress. Regarding the target 4UFD (Thrombin), pedunculagin stabilizes an inactive conformation of the enzyme by interacting with critical residues such as His57, Asp189, and Ser195 through hydrogen bonds and hydrophobic interactions, thereby disrupting the coagulation cascade. For the target 3W37 (Alpha-Glucosidase), pedunculagin blocks the active site by forming hydrogen bonds with Asp352 and Glu411, thereby inhibiting carbohydrate digestion and contributing to blood sugar regulation. Regarding 5FSA (CYP51 Variant1), pedunculagin binds to the enzyme’s active site, interacting with catalytic residues such as Tyr118 and His310, disrupting ergosterol biosynthesis and altering fungal cell viability. Finally, for the target 4DX5 (Acriflavine Resistance Protein B), pedunculagin interacts with periplasmic residues such as Gln125 and Asp408, stabilizing the T conformation and inhibiting the bacterial efflux mechanism. These detailed interactions, involving specific residues and well-defined reaction mechanisms, confirm the versatile potential of pedunculagin as an antibacterial, antifungal, antioxidant, antidiabetic, and antithrombotic agent, paving the way for in-depth experimental studies to validate these mechanisms and explore its therapeutic applications.

**TABLE 12:** Pedunculagin’s molecular interactions with target proteins.

Targets	2D	Interaction 3D
2CDU	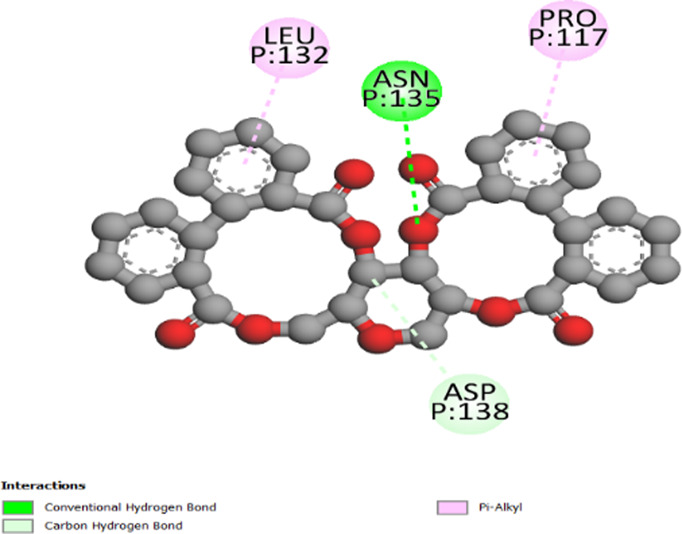	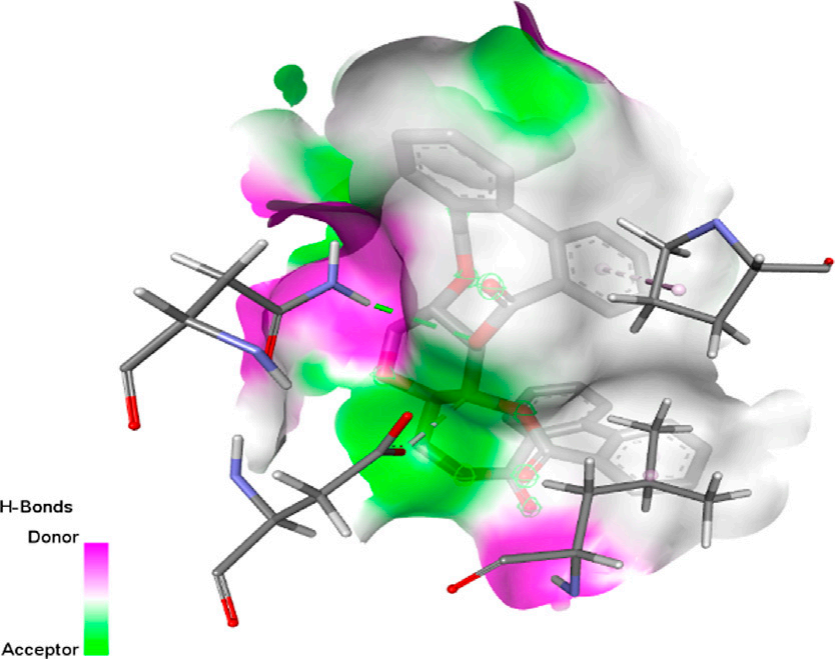
3W37	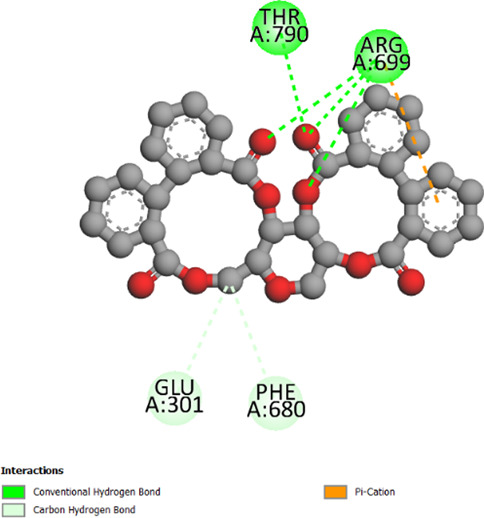	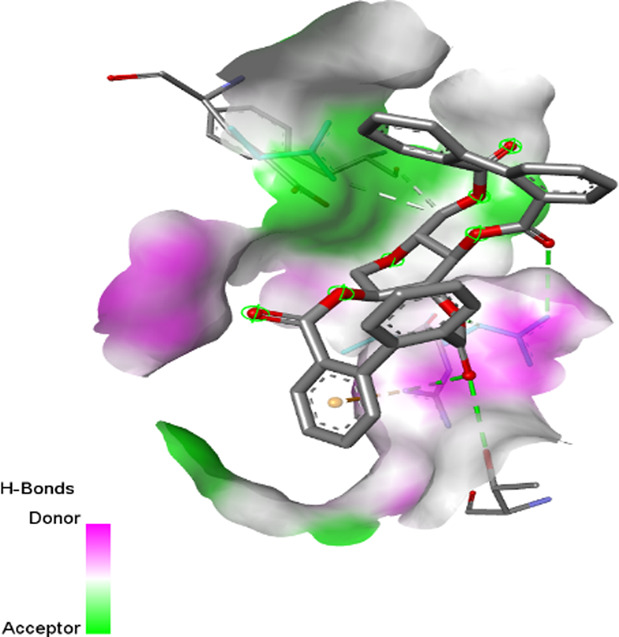
4UFD	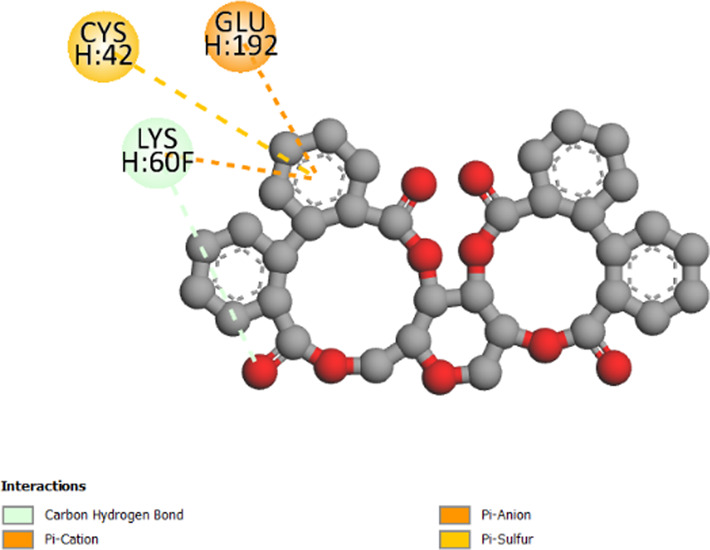	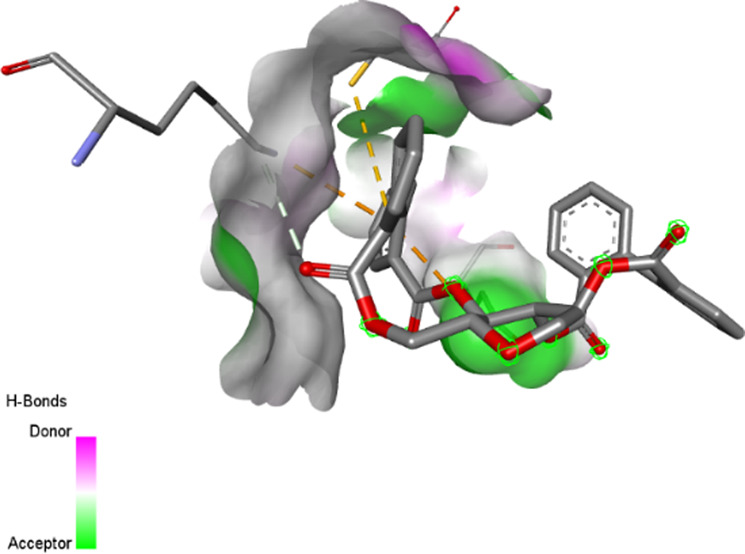
4W93	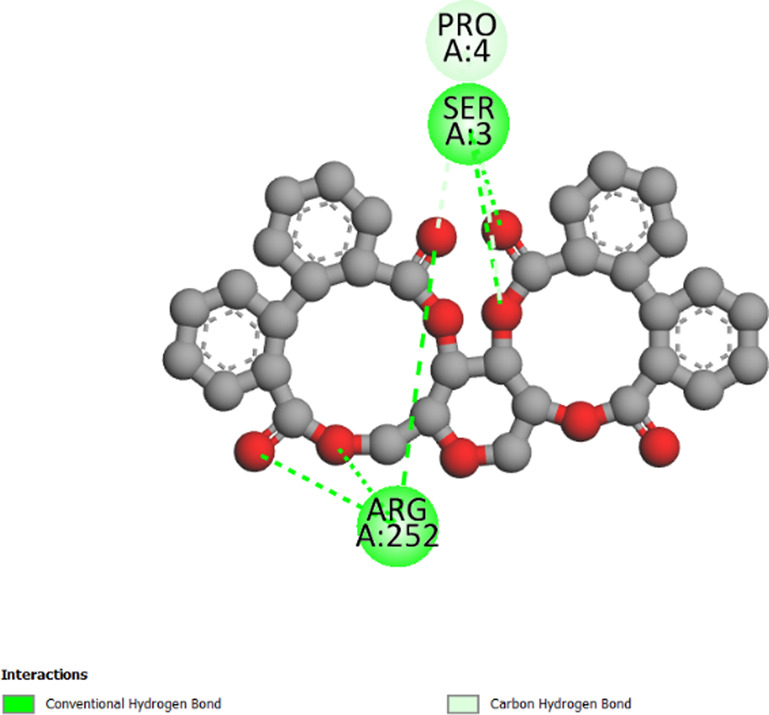	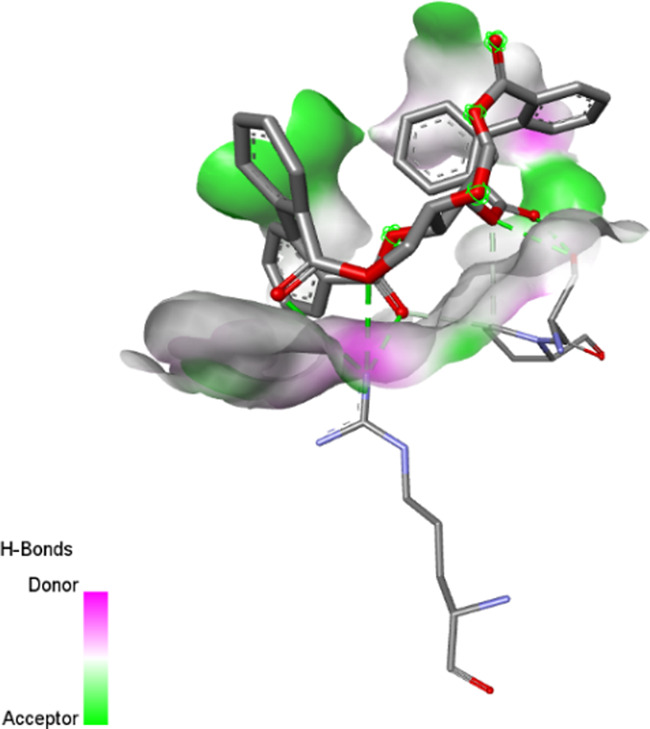
5FSA	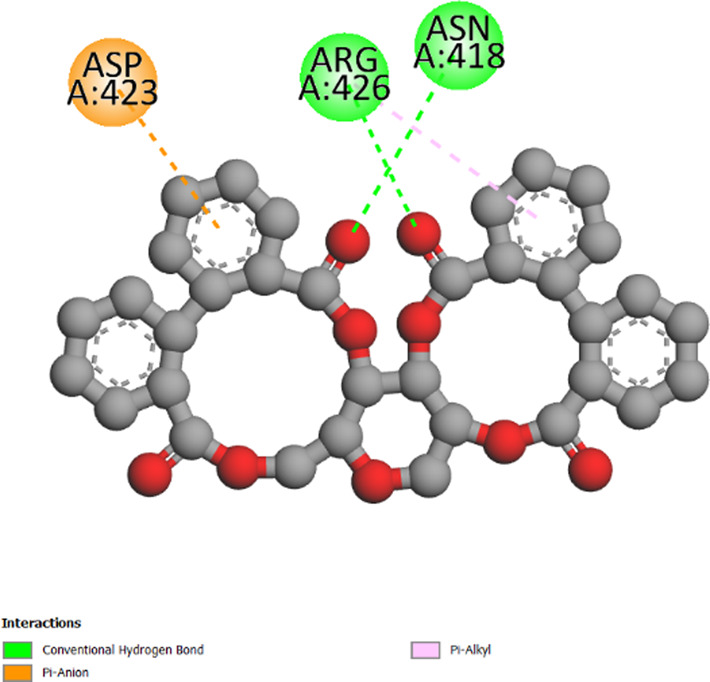	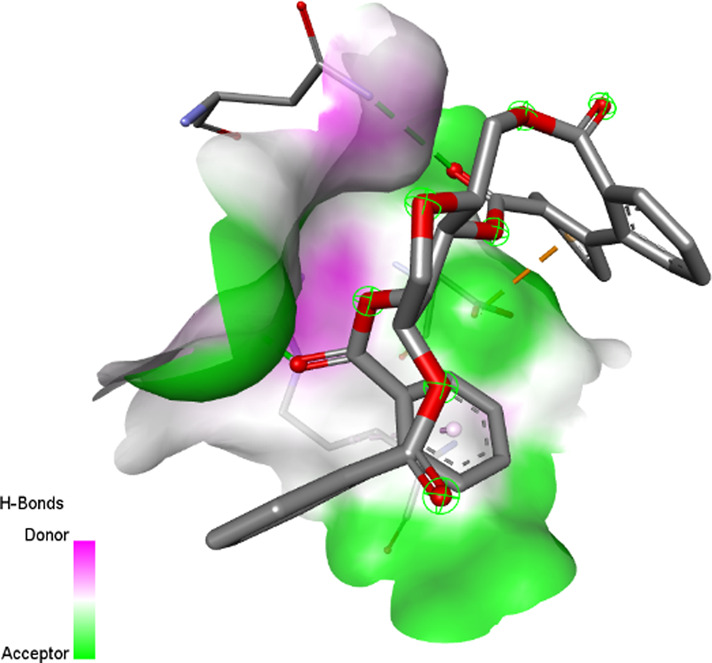

### 3.9 Molecular dynamics simulation

Molecular dynamics simulations were conducted on the docked complexes of a lead molecule, identified via molecular docking studies, demonstrating strong binding affinity for all target proteins. The simulations assessed the stability, conformational dynamics, and interaction patterns of the molecule-protein complexes in physiological conditions.

#### 3.9.1 RMSD analysis

**FIGURE 12:**
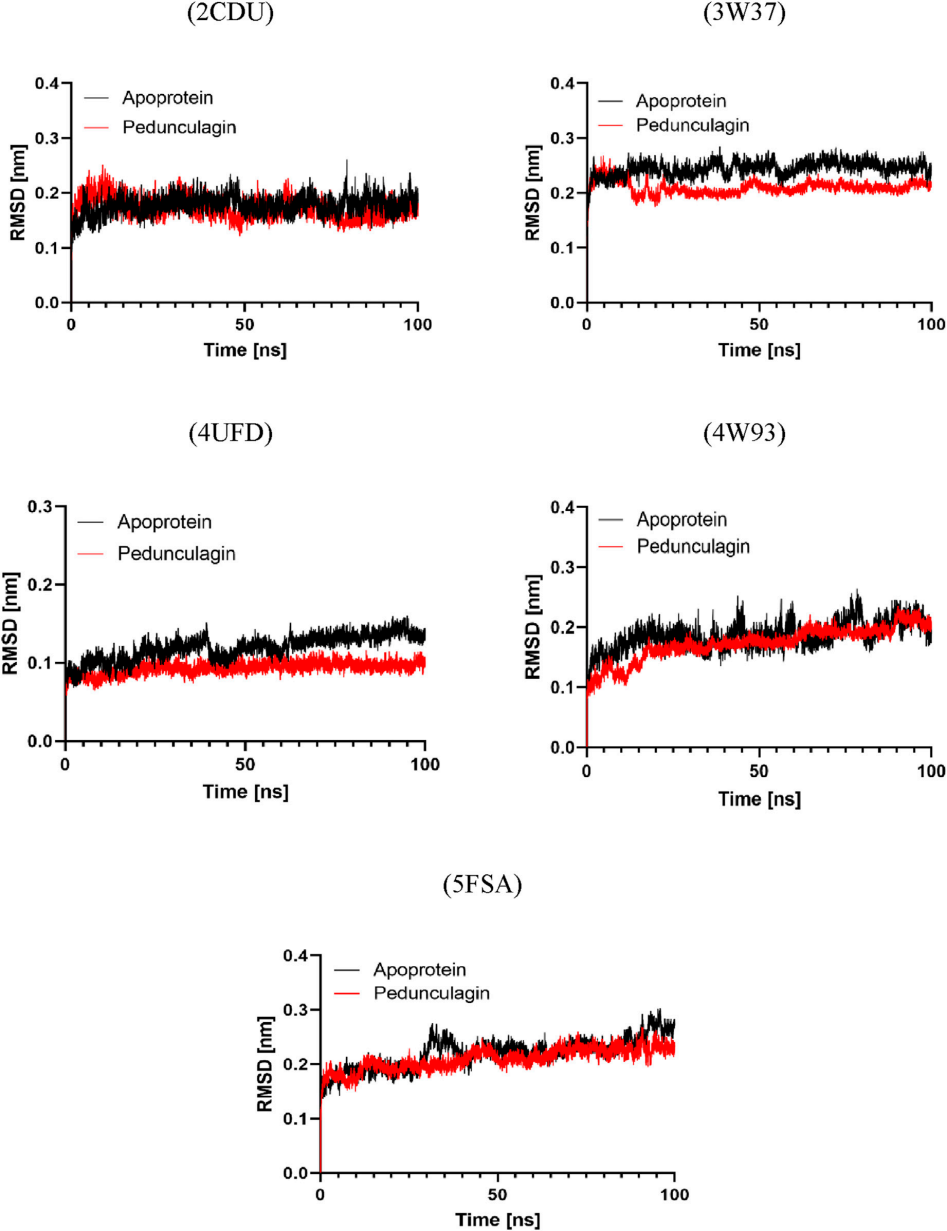
RMSD profile analysis of Pedonculagin complexed with diverse protein targets: 2CDU, 3W37, 4UFD, 4W93, and 5FSA.

An analysis of the root mean square deviation (RMSD) was performed to assess the protein-ligand complexes’ structural stability relative to the target proteins’ apo forms. The findings indicated that the Pedonculagin-protein complexes consistently demonstrated lower RMSD values than the apoproteins across all targets. Figure 12 illustrates that the RMSD profiles indicate Pedonculagin binding plays a role in stabilizing protein structures, thereby diminishing conformational fluctuations. The decreased RMSD in the complexes indicates that Pedonculagin effectively interacts with the target proteins and improves their structural integrity, positioning it as a candidate for further study. RMSD analysis alone does not adequately elucidate specific protein regions’ localized flexibility and dynamic behavior. The Root Mean Square Fluctuation (RMSF) was computed to overcome this limitation, facilitating a residue-specific examination of flexibility and mobility. The integration of RMSF results facilitated a more nuanced understanding of the impact of Pedonculagin on protein flexibility.

#### 3.9.2 RMSF analysis

**FIGURE 13:**
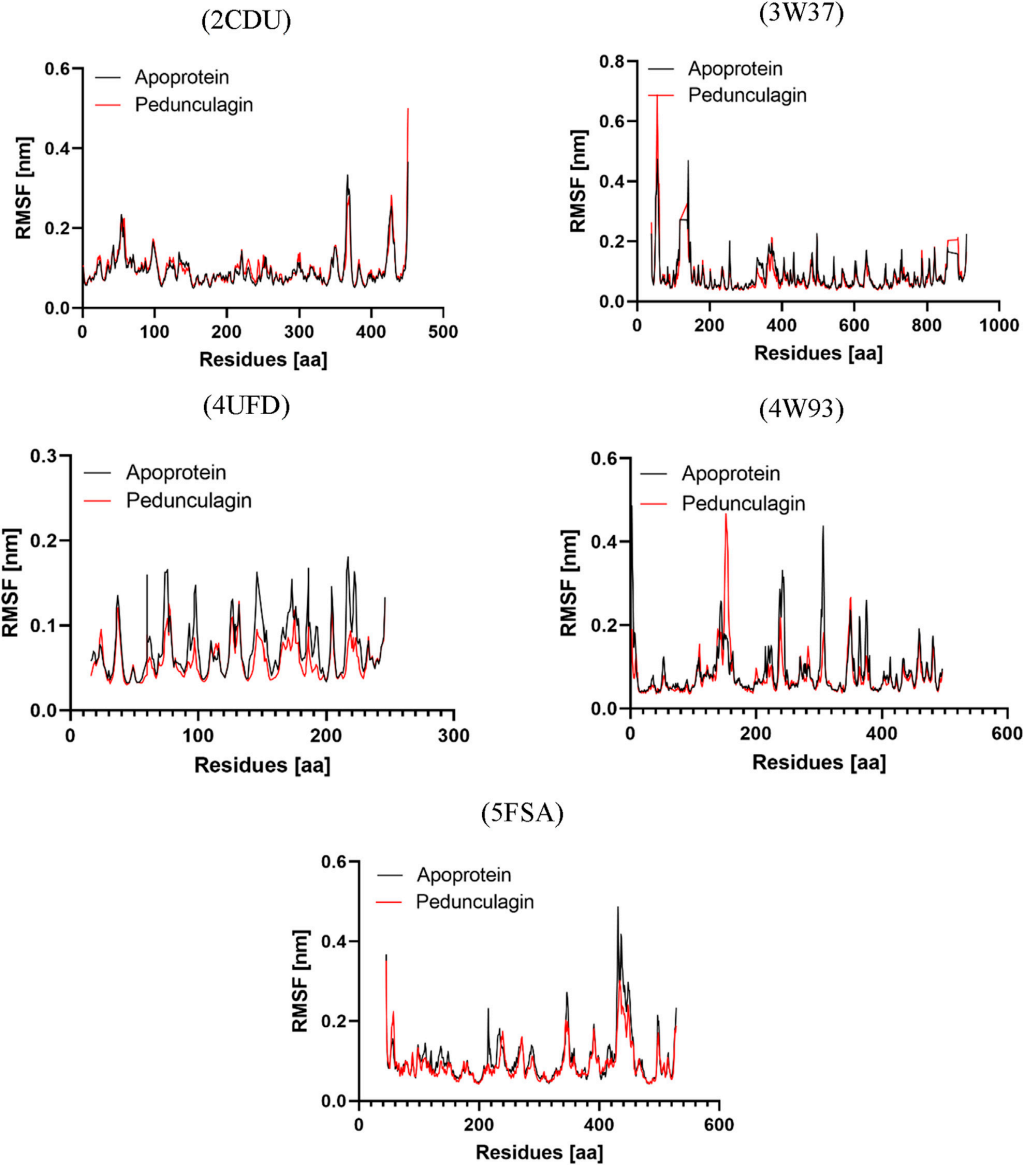
RMSF profile analysis of Pedonculagin complexed with diverse protein targets: 2CDU, 3W37, 4UFD, 4W93, and 5FSA.

The Root Mean Square Fluctuation (RMSF) analysis was performed to investigate the apo proteins’ residue-level flexibility and dynamic behavior and their respective complexes with Pedonculagin across all target proteins. As shown in Figure 13, the results demonstrated that the Pedonculagin-protein complexes exhibited consistently lower RMSF values compared to the apoproteins, indicating reduced residue-level fluctuations upon ligand binding. This suggests that Pedonculagin stabilizes the overall protein structure, as shown by RMSD, and minimizes local flexibility, particularly in key binding regions and other dynamic areas of the proteins. The decreased RMSF values highlight the potential of Pedonculagin to enhance protein rigidity in a functionally favorable manner, further supporting its role as a stabilizing agent and a promising therapeutic candidate.

#### 3.9.3 Radius of gyration analysis

**FIGURE 14:**
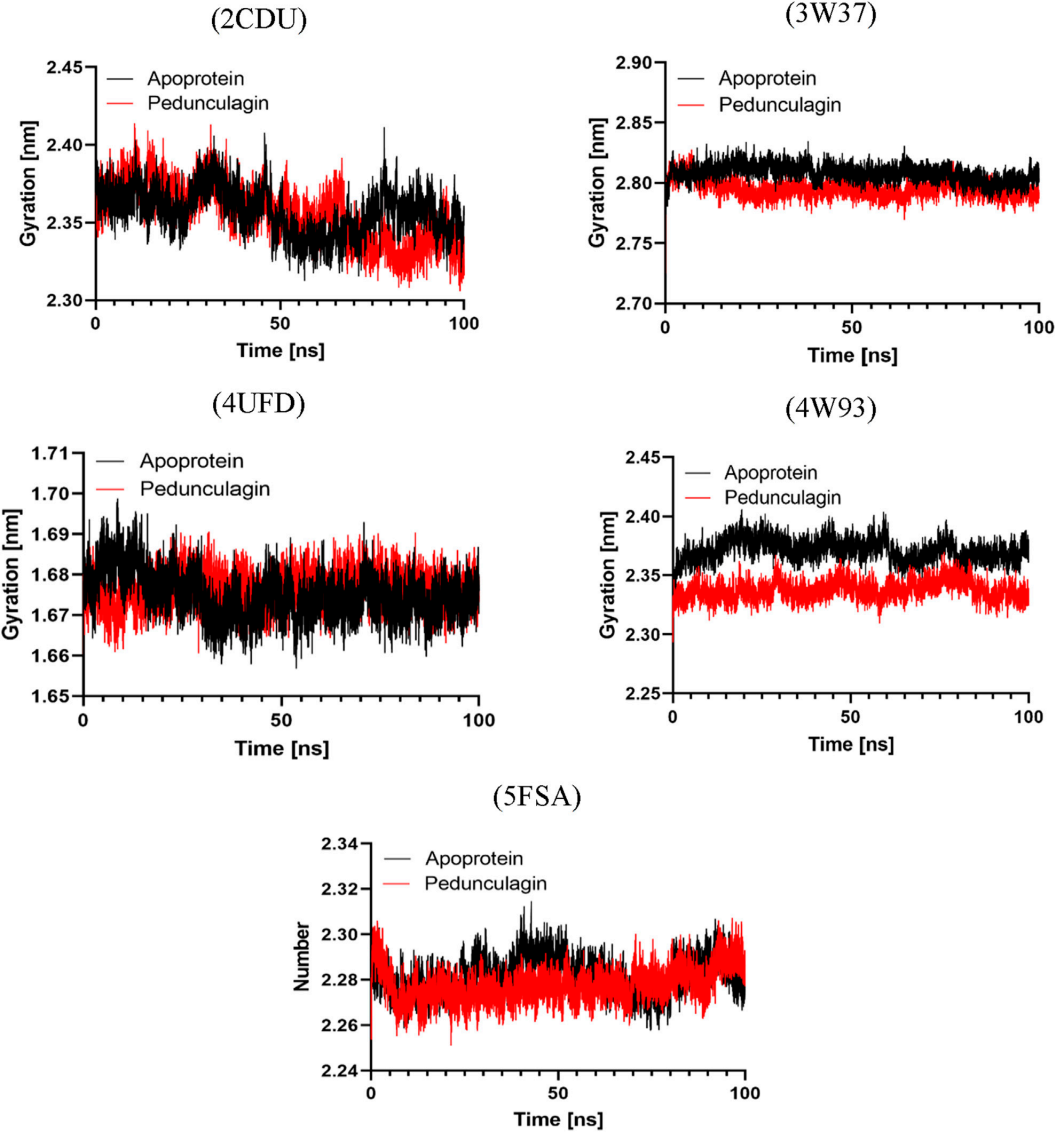
Radius of Gyration profile analysis of Pedonculagin complexed with diverse protein targets: 2CDU, 3W37, 4UFD, 4W93, and 5FSA.

To further assess the structural compactness and stability of the Pedonculagin-protein complexes, the Radius of Gyration (Rg) was calculated and compared with the apo proteins for each target. As shown in Figure 14, the results indicated that the Pedonculagin-protein complexes consistently exhibited lower Rg values than the apo forms. This suggests that the binding of Pedonculagin leads to a more compact and stable protein structure, reducing overall structural dispersion. The lower Rg values align with the RMSD and RMSF findings, further confirming that Pedonculagin enhances the stability and rigidity of the protein targets. These results reinforce the potential of Pedonculagin to act as a stabilizing ligand with promising implications for therapeutic applications.

#### 3.9.4 H-bond analysis

**FIGURE 15:**
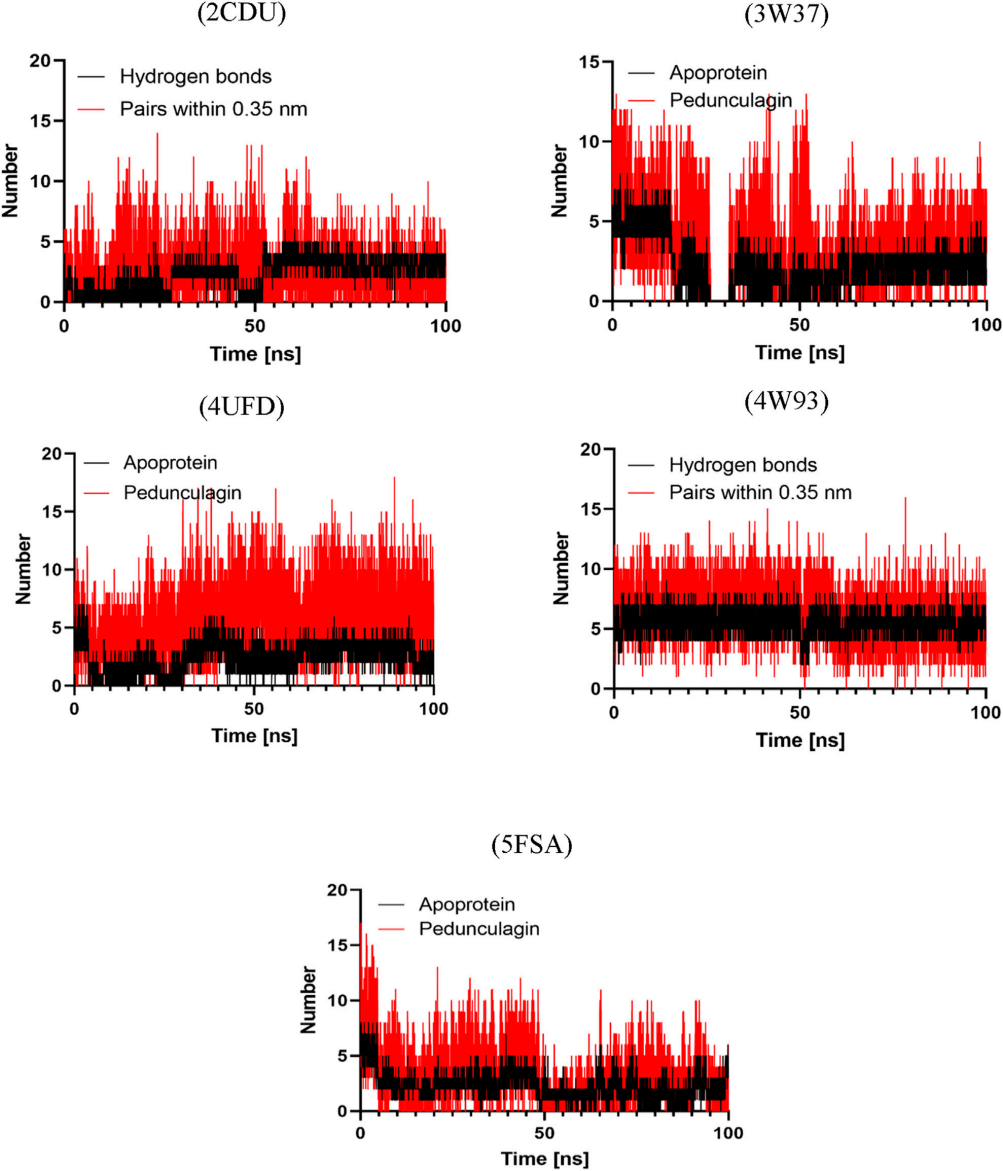
Hydrogen Bond Analysis of Pedonculagin complexed with diverse protein targets: 2CDU, 3W37, 4UFD, 4W93, and 5FSA.

Hydrogen bonding plays a pivotal role in determining the binding affinity and stability of ligand–protein complexes, profoundly influencing drug specificity, absorption, and metabolism (Böhm and Schneider, 2006). To further validate the stability of the docked complexes, hydrogen bond formation between Pedongulagin and the selected protein targets (2CDU, 3W37, 4UFD, 4W93, and 5FSA) was monitored under solvent conditions throughout molecular dynamics (MD) simulations (Figure 15). The results demonstrated that Pedongulagin consistently formed stable hydrogen bond interactions within the active sites of all targets. Specifically, it exhibited an average of 2.195 hydrogen bonds with 2CDU, 2.141 with 3W37, and 2.172 with 5FSA, while a slightly higher average of 2.393 hydrogen bonds was observed with 4UFD, suggesting enhanced stability at this binding site. Notably, the Pedongulagin–4W93 complex showed the highest number of hydrogen bonds, averaging 5.33, indicating a particularly robust and stable interaction. These findings underscore the crucial role of hydrogen bonding in mediating the stability of Pedongulagin–protein interactions and highlight Pedongulagin’s potential as a promising multitarget inhibitor with strong binding affinity and favorable interaction stability, making it a valuable candidate for further therapeutic development.

### 3.10 MM-PBSA binding free energy analysis

Binding free energy calculations were conducted using the MmPbSaStat.py script from the g_mmpbsa package, which utilizes output files from MM-PBSA simulations to compute the average binding free energy and the associated standard deviation across the entire molecular dynamics (MD) trajectory (Dasmahapatra et al., 2022) (Table 13). This approach allowed for a more detailed evaluation of the binding stability and interaction profiles of the selected ligand–protein complexes.

**TABLE 13:** MM-PBSA calculations of binding free energy for all the complexes.

Complex	Binding energy	SASA energy	Polar solvation	Electrostatic	Van der waals
	(kJ/mol)	(kJ/mol)	Energy (kJ/mol)	Energy (kJ/mol)	Energy (kJ/mol)
Pedongulagin- 2CDU	−30.820 ± 13.395	−17.947 ± 0.977	239.541 ± 17.711	−122.682 ± 16.441	−129.732 ± 14.532
Pedongulagin - 3w37	−30.788 ± 24.687	−10.263 ± 1.362	138.154 ± 33.604	−64.208 ± 14.358	−94.471 ± 11.878
Pedongulagin -4UFD	−30.799 ± 19.793	−18.401 ± 1.357	231.003 ± 26.918	−93.108 ± 23.979	−150.293 ± 13.020
Pedongulagin - 4W93	−59.555 ± 14.484	−19.494 ± 1.624	293.966 ± 18.359	−202.749 ± 15.175	−131.277 ± 21.641
Pedongulagin - 5FSA	−19.719 ± 19.096	−12.086 ± 1.937	164.843 ± 58.108	−79.433 ± 47.89	−93.043 ± 16.518

The MM-PBSA analysis revealed that Pedongulagin forms consistently favorable interactions with all five protein targets, with binding free energies ranging from −19.72 to −59.56 kJ/mol. These results suggest that Pedongulagin has the potential to act as a stable and versatile binder across multiple targets. Notably, the Pedongulagin–4W93 complex exhibited the strongest binding affinity (−59.56 ± 14.48 kJ/mol), predominantly driven by strong electrostatic interactions (−202.75 ± 15.18 kJ/mol) and significant van der Waals contributions (−131.28 ± 21.64 kJ/mol), which effectively counterbalance the polar solvation penalty.

Similarly, Pedongulagin also showed substantial binding affinities with 2CDU, 4UFD, and 3W37, with binding free energies around −30.8 kJ/mol, resulting from balanced contributions of van der Waals and electrostatic interactions. While the 5FSA complex demonstrated the weakest binding energy (−19.72 ± 19.10 kJ/mol), it still maintained meaningful non-covalent interactions, highlighting Pedongulagin’s retained binding capacity even under less favorable energetic conditions. Overall, van der Waals forces consistently contributed to complex stabilization across all systems, while electrostatic interactions played a critical role in enhancing the binding strength in top-performing complexes. Collectively, these findings underscore Pedongulagin’s strong binding potential, with the 4W93 complex emerging as the most promising for further experimental validation.

## 4 Discussion

The quality control findings for *J. regia* affirm its potential as an essential source for both nutraceutical and pharmacological applications. Its low moisture content, in alignment with international standards (FAO, 2021), ensures optimal preservation while minimizing the risks of microbial degradation, which is vital for maintaining the stability of plant-based products. The pH, which is close to neutral, further supports its chemical stability, and its high mineral content highlights its nutritional importance, as evidenced by the study conducted by Song et al. (Song et al., 2022) on *Juglans* species. Additionally, the levels of heavy metals comply with the FAO/WHO standards (2009), ensuring product safety, a key factor in using medicinal and food plants (World Health Organization, 2020). These results align with the research of Ncube et al. (Ncube et al., 2012), which underscores the influence of environmental factors on plant quality. *Juglans regia* stands out for its chemical stability, mineral composition, and adherence to safety regulations, enhancing its suitability for various applications.

The phytochemical analysis of *J. regia* husks reveals a diverse array of bioactive secondary metabolites, highlighting its potential for both pharmacological and nutraceutical applications. Steroids, triterpenes, and saponins are notably abundant and are recognized for their anti-inflammatory and immunomodulatory properties, as reported by Bhat et al. (2023). Flavonoids and tannins, present in moderate quantities, contribute to the antioxidant and antimicrobial effects, as supported by the studies of Carvalho et al. (2010), Mosaddad et al. (2023), Quratul-ain and Siddiqui, (2024). These compounds have been extensively investigated for their cardioprotective, neuroprotective, and lipid oxidation-inhibiting effects, which underpin the growing interest in their application in pharmaceutical and agro-food formulations (Croitoru et al., 2019; Mateş et al., 2024). The absence of sugars, holosides, and alkaloids points to a distinctive chemical profile, which may offer reduced toxicity advantages, as Vezikov and Simpson suggested (Vezikov and Simpson, 2025). Furthermore, the analysis of extraction yields demonstrates that decoction is the most efficient method for extracting polyphenols and flavonoids, outperforming Soxhlet extraction due to the prolonged exposure to hot water, as noted by Shrivastav et al. (2024). On the other hand, Soxhlet extraction with water shows superior efficiency in extracting tannins, reflecting the varying solubility of phenolic compounds.

The Moroccan walnut husks examined in this study exhibit high concentrations of phenolic compounds, with polyphenol content ranging from 26.204 to 42.105 mg GAE/g of extracts, surpassing the values reported by Leahu et al. (2016) for Romanian walnuts (17.28-23.52 mg GAE/g). Flavonoid levels are higher than those identified by Sharma et al. (2014), which were 12.88 mg QE/g, although slightly lower than the 22.91 mg QE/g reported by Ghasemi et al. (2011). The concentrations of both condensed and hydrolysable tannins correspond with the findings of Walnut (Qiang, 2015), Meshkini and Tahmasbi, (2017), and Slatnar et al. (2015). The analysis highlights the significant impact of the extraction method on the levels of secondary metabolites. Decoction emerges as the most efficient technique for extracting polyphenols and flavonoids, as it facilitates the solubilization of these compounds through an extended thermal process, as noted by Dai and Mumper (2010) and Zhao et al. (Dai and Mumper, 2010; Zhao et al., 2019). Conversely, tannins are more effectively extracted using the Soxhlet method with water due to their aqueous solubility under reflux, as demonstrated by Aspé and Fernández (2011), Hoyos-Martínez et al., and Markom et al. (Markom et al., 2010; Aspé and Fernández, 2011; De Hoyos-Martínez et al., 2019). Hydroethanolic maceration yields lower quantities of these compounds, as observed by Paşayeva et al. (2025). These findings underscore the importance of extraction conditions in optimizing the recovery of bioactive compounds, highlighting opportunities for the valorization of walnut husks in nutraceutical and pharmacological applications, particularly due to the antioxidant, cardioprotective, and antimicrobial properties of polyphenols and tannins.

The HPLC/MS analysis of *J. regia* husks reveals a notable diversity of bioactive compounds, predominantly polyphenols, flavonoids, and phenolic acids, primarily responsible for its pharmacological and nutraceutical properties. Pedunculagin, a hydrolyzable tannin recognized for its antioxidant and antimicrobial effects, constitutes a significant fraction among these compounds. Additionally, hydrojuglone glucoside, regiolone, and gallotannin are identified, confirming the phenolic richness of the husks, as reported by Medic et al. (2021), Snarska et al. (2024), and Yin et al. (2015). The fragmentation mechanisms, specifically the loss of galloyl groups and the cleavage of glycosidic bonds, reveal the structural complexity of these metabolites. The husks exhibit a higher concentration of hydrolysable tannins than the leaves and other husks, which aligns with the findings of Jahanban-Esfahlan et al. (2019a). Moreover, the phenolic acids identified, including gallic acid, chlorogenic acid, and ferulic acid, show concentrations comparable to those observed in other *Juglans* species, thus confirming a consistent phytochemical profile, as noted by Bourais et al. (2022), Croitoru et al. (2019), Jahanban-Esfahlan et al. (2019a). These results highlight the potential of *J. regia* husks for antioxidant, antimicrobial, antithrombotic, and anti-diabetic applications, properties that are well-documented for tannins and phenolic acids.

The evaluation of the antioxidant properties of aqueous and hydroethanolic extracts of *J. regia* reveals a notable ability to capture free radicals and reduce oxidative species, reinforcing their potential as natural antioxidants. The aqueous decoction (E0) stands out due to its high levels of polyphenols and flavonoids, compounds known for their ability to stabilize free radicals, as demonstrated by Zhang et al. (2009) and Jahanban-Esfahlan et al. (2019b). The results of the DPPH and FRAP assays indicate significant antioxidant activity in the aqueous extract. In contrast, the hydroethanolic extract (E2) performs superior in the TAC test, suggesting that hydrophobic compounds, likely specific tannins, play a significant role in this activity. *Juglans regia* extracts exhibit promising antioxidant activity, although slightly less potent than ascorbic acid, in agreement with Milić et al. (2024) on natural antioxidants. This variation highlights the complex interactions among the diverse bioactive compounds involved. The discrepancies observed in the DPPH, FRAP, and TAC results emphasize the importance of using a multi-method approach to evaluate antioxidant mechanisms. These methods capture various facets of antioxidant activity, such as free radical reduction and metal chelation, as noted by Munteanu and Apetrei, (2021).

Walnuts are distinguished by their superior antioxidant activity, surpassing that of other nuts due to their high content of phenolic compounds, including hydrolyzable tannins, tocopherols, and melatonin. These findings support the conclusions of Jabli et al. (2017), which indicated that aqueous-organic extracts of whole walnuts contain significant levels of total phenolic compounds and exhibit remarkable antioxidant capacity, as evidenced by an IC_50_ of 343 μg/mL in the DPPH assay. The findings of this study demonstrate superior antioxidant activity compared to previous reports in the literature. In the survey conducted by Jiong et al. (2014), the ethyl acetate extract exhibited the highest radical scavenging capacity, effectively neutralizing DPPH, hydroxyl, and superoxide radicals, with a CE_50_ value of 81.03 μg/mL, followed by the methanol extract (CE_50_ = 131.35 μg/mL) and the chloroform extract (CE_50_ = 176.35 μg/mL). Notably, our results outperform those reported by Cerit et al. (2017), which documented FRAP antioxidant capacity values ranging from 156 to 302 mg/g. These comparisons highlight the notable efficacy of the *J. regia* decocted extract as a potent natural antioxidant, further supporting its potential therapeutic and nutraceutical applications. Kornsteiner et al. (2006) emphasized the importance of the defatted fraction of walnuts, which accounts for most of the antioxidant activity. Recent studies have extended this analysis by assessing the antioxidant activity of various components of the walnut fruit, including the husks, shell, defatted kernel, leaves, and stems, using multiple spectroscopic techniques such as TAA, RP, FRAP, and ORAC. Despite the challenges posed by heterogeneous measurement units, these studies reveal that the shell contains the highest concentration of phenolic compounds, with an ORAC value of 3423.44 ± 142.52 μmol TE/g. These findings confirm the potential of nuts, especially their various fractions, as a source of natural antioxidants.

A significant correlation was observed between the concentrations of phenolic compounds and their antioxidant efficacy. Polyphenols exhibit negative correlations with the FRAP and TAC tests, potentially due to specific interactions or the influence of non-phenolic compounds. Tannins, both condensed and hydrolysable, show a strong correlation (r = 0.98), highlighting their crucial role in antioxidant mechanisms. These observations are consistent with research conducted by Yin et al. (2015) and Żurek et al. (2022), which indicated that *J. regia* extracts, rich in flavonoids, gallic acid, and hydrolysable tannins, demonstrate significant antioxidant activity, particularly in metal chelation assays. The results suggest a synergistic effect among polyphenols, flavonoids, and tannins, functioning through complementary mechanisms.

The evaluation of the antimicrobial properties of the decoction extract of *J. regia* reveals significant efficacy against various pathogenic microorganisms, although typically less potent than standard antibiotics and antifungals. Gram-positive bacteria, particularly *Enterococcus faecalis* (MIC = 300 μg/mL), show heightened sensitivity to the extract, which aligns with previous research on the antibacterial properties of polyphenols and tannins derived from *J. regia* (D’Angeli et al., 2021). In contrast, the extract’s effectiveness against methicillin-resistant *Staphylococcus aureus* (MRSA) is somewhat limited (MIC = 5000 μg/mL), despite the ability of phenolic compounds to interfere with biofilms and membrane permeability, as highlighted by Carrillo (Carrillo, 2020). The extract exhibits targeted activity against *Acinetobacter baumannii* and *Shigella* sp. (MIC = 150 μg/mL) in Gram-negative bacteria, suggesting that compounds such as flavonoids and quinones may alter the cell wall or inhibit essential enzymes, as indicated by Jubair et al. (2021). Strains like *Escherichia coli* and *Klebsiella pneumoniae* show elevated MICs (>5000 μg/mL), which may be attributed to the complexities of their outer membrane, potentially hindering the extract’s efficacy. The *J. regia* extract demonstrates moderate antifungal activity against *Candida albicans* and *Aspergillus niger*, with a minimum inhibitory concentration (MIC) ranging from 2,500 to 5000 μg/mL, which is lower than that of terbinafine. Nevertheless, the extract may serve as a supplementary treatment, particularly due to the presence of naphthoquinones like hydrojuglone and ruginone, which are known for their ability to induce oxidative stress and compromise cell membranes, as noted by Ahmad and Suzuki (2019) and Li et al. (2024). The findings are consistent with studies by Arslan et al. (2023) and Oliveira et al. (2008), which demonstrated comparable effectiveness against Gram-positive bacteria and specific yeasts, underscoring the role of extraction conditions in antimicrobial activity. The husks of *J. regia* represent a promising source of natural antimicrobial compounds, particularly against resistant strains, thereby increasing their potential for developing complementary treatments using natural products.

The investigation of the anticoagulant properties of *J. regia* reveals a significant, dose-dependent prolongation of coagulation times, suggesting its potential application in natural thromboprophylaxis. At a concentration of 11.5 mg/mL, the extract extends the prothrombin time (PT) to 98.9 s and the activated partial thromboplastin time (PTT) to 134.2 s, notably higher than the low doses of heparin, where PT ranges from 13.4 to 14.1 s. These results indicate substantial anticoagulant activity, likely associated with the modulation of coagulation factors, although the precise mechanism remains fully elucidated. The findings are consistent with the research by Rywaniak et al. (2015) and Bojić et al. (2019), which linked the anticoagulant properties of *J. regia* to its polyphenols and flavonoids, which inhibit thrombin and fibrinogen. Jahanban-Esfahlan et al. (2019b) and Amirou et al. (2023) demonstrated that specific phenolic compounds in *J. regia* chelate calcium ions, leading to a reduction in the activity of calcium-dependent coagulation factors (factors II, VII, IX, and X). In comparison to other anticoagulant plants like garlic (*Allium sativum*) and ginger (*Zingiber officinale*), *J. regia* shows more pronounced effects at higher concentrations, suggesting a specific interaction with coagulation proteins, as noted by Lamponi, (2021). Unlike heparin, which primarily acts through antithrombin III, *J. regia* may involve additional mechanisms, such as direct inhibition of coagulation factors or modulation of oxidative stress, a key element in platelet aggregation. The dose-dependent response observed further supports the pharmacological potential of *J. regia*; however, further research into its bioavailability, drug interactions, and side effects is necessary to confirm its clinical relevance. Consequently, the Moroccan samples of *J. regia* appear promising for thromboembolic disease prevention, offering a natural alternative to synthetic anticoagulants, pending further studies to validate their safety and efficacy.

The evaluation of the antidiabetic activity of the decocted extract of *J. regia* reveals a dose-dependent inhibition of the digestive enzymes α-amylase and α-glucosidase, supporting its potential to regulate postprandial hyperglycemia. In this context, the extract demonstrates superior efficacy to acarbose for inhibiting α-amylase (EC_50_ = 104.798 μg/mL *versus* 364.446 μg/mL) and a potent inhibition of α-glucosidase, with a lower EC_50_ (12.12 μg/mL *versus* 17.27 μg/mL for acarbose). These results suggest that the polyphenols and hydrolyzable tannins in *J. regia* effectively interact with these enzymes, thereby reducing carbohydrate absorption and minimizing glycemic fluctuations. These findings are consistent with the research by Wang et al. (2020) and Farazi et al. (2024), which demonstrated that the phenolic compounds in *J. regia* inhibit these enzymes by forming complexes with their active sites. Recent studies further confirm that the inhibitory effect of polyphenols on α-amylase and α-glucosidase increases with the number of galloyl groups they contain, as noted by Visvanathan et al. (2024). Among the polyphenols found in nuts, compounds such as casuarinin and pedunculagin are particularly noted for their strong ability to inhibit these enzymes. Given their high bioaccessibility after digestion (Li et al., 2023), these polyphenols could explain the health benefits of walnuts, particularly in regulating digestive enzymes and reducing postprandial glucose spikes, as suggested by Balakrishna et al. (2022). *In vivo*, as tested in our study, the decocted extract at 400 mg/kg significantly reduces postprandial blood glucose in rats, even surpassing glibenclamide, a reference antidiabetic. This effect is linked to reduced blood glucose rise and improved carbohydrate metabolism modulation, as also noted by Afra et al. (2023). This mechanism may be related to stimulating insulin secretion, improving insulin sensitivity, and inhibiting intestinal glucose absorption—standard mechanisms in polyphenol-rich extracts, as demonstrated by Domínguez Avila et al. (2017). Moreover, the extract shows no acute toxicity, even at high doses (2 g/kg), enhancing its safety profile compared to synthetic antidiabetic drugs, which are often associated with side effects. Compared to other hypoglycemic plants, such as *Momordica charantia* (Leung et al., 2009) or *Cinnamomum verum* (Wariyapperuma et al., 2020), *J. regia* offers comparable, if not superior, efficacy, with better tolerance. However, further studies are necessary to clarify the underlying molecular mechanisms, particularly the effects on insulin signaling and glucose transporters (GLUT4), and to confirm its efficacy and safety in human clinical trials.

The docking and molecular dynamics analysis of bioactive compounds from *J. regia* reveals significant binding affinity and structural stabilization of protein-ligand complexes, reinforcing the pharmacological activities observed in both *in vitro* and *in vivo* studies. Pedunculagin and hydrojuglone glucoside, key compounds from *J. regia* husks, demonstrate strong interactions with essential proteins associated with antibacterial, antifungal, antioxidant, antidiabetic, and antithrombotic activities. Pedunculagin exhibits a notable affinity for the acriflavine resistance protein (4DX5, -10.1 kcal/mol) and thrombin (4UFD, −8.9 kcal/mol), which supports the experimentally observed antibacterial and anticoagulant activities. Similarly, hydrojuglone glucoside interacts with D-alanine-D-alanine ligase (2CAG, −9.6 kcal/mol) and NADPH oxidase (2CDU, −9.2 kcal/mol), suggesting its potential to inhibit bacterial cell wall biosynthesis and reduce oxidative stress, in line with *in vitro* results. These interactions provide insight into the antifungal activity, indicating that the compounds may inhibit lanosterol 14-alpha demethylase (5FSA, −8.0 kcal/mol), an essential enzyme in ergosterol biosynthesis, thereby confirming their effects against *C. albicans* and *A. niger*. The simulations also show that these compounds effectively inhibit α-amylase (4W93, -8.3 kcal/mol) and α-glucosidase (3W37, -8.0 kcal/mol), which correlates with the observed reduction in postprandial blood glucose levels *in vivo*. The findings are consistent with both *in vitro* and *in vivo* studies, which attribute these effects to polyphenols and hydrolysable tannins. Molecular interactions, including hydrogen bonds with Arg91 and Tyr198 in NADPH oxidase (2CDU) and with His57, Asp189, and Ser195 in thrombin (4UFD), shed light on the mechanisms underlying antioxidant and anticoagulant activity. Molecular dynamics simulations further show that these compounds stabilize protein-ligand complexes, as evidenced by lower RMSD and RMSF values, increased compaction (reduced Rg), and persistent hydrogen bonds. These results suggest the rigidification of functional regions and an enhancement in structural integrity. The findings indicate that pedunculagin acts as a stabilizing agent, limiting the conformational flexibility of proteins and extending their active state, which enhances its therapeutic potential for antimicrobial, antioxidant, antidiabetic, and antithrombotic applications. However, further experimental studies are necessary to confirm these *in silico* predictions and to assess the bioavailability and kinetic actions of these compounds in actual physiological conditions, thus paving the way for novel pharmacological applications derived from *J. regia*.

## 5 Conclusion

Regarded as a significant natural resource, *J. regia* is distinguished by its physico-chemical stability and a rich composition of bioactive compounds, making it highly relevant for innovative applications in human health. According to international standards (FAO/WHO), its chemical profile is characterized by low moisture, neutral pH, and a balanced mineral composition, which ensures optimal preservation and safety, rendering it suitable for pharmaceutical and nutraceutical applications. The presence of secondary metabolites, particularly polyphenols, hydrolysable tannins, and flavonoids, contributes to its antioxidant, anticoagulant, and antidiabetic properties, which in some cases may surpass those of conventional pharmaceuticals. Molecular docking and *in silico* dynamics studies reveal the targeted interactions of pedunculagin and hydrojuglone glucoside with key proteins, thereby enhancing their therapeutic potential against microbial infections, hyperglycemia, and thrombotic disorders. Extraction techniques, particularly decoction, improve the recovery of these bioactive compounds, and the absence of acute toxicity at higher doses further supports its safety profile. The findings suggest that *J. regia* could serve as a promising natural alternative to synthetic treatments, particularly for conditions like type 2 diabetes and cardiovascular diseases. Clinical studies are essential to validate bioavailability, understand the mechanisms of action *in vivo*, and assess the long-term effectiveness of *J. regia*. This plant represents a bridge between traditional knowledge and modern innovation, offering sustainable and personalized therapeutic solutions. Its integration into nutraceutical or pharmaceutical formulations has the potential to address pressing public health challenges while promoting the responsible use of botanical resources.

## Data Availability

The original contributions presented in the study are publicly available. This data can be found here: https://doi.org/10.5281/zenodo.16893990.
